# Molecular subtypes based on cuproptosis-related genes and tumor microenvironment infiltration characterization in ovarian cancer

**DOI:** 10.1186/s12935-022-02756-y

**Published:** 2022-10-28

**Authors:** Jingjing Zhang, Miao Lu, Haoya Xu, Fang Ren, Liancheng Zhu

**Affiliations:** Department of Obstetrics and Gynecology, Shengjing Hospital of China Medical University, Shenyang, Liaoning China

**Keywords:** Cuproptosis, Ovarian cancer, Molecular subtype, Tumor microenvironment, Prognosis, Immune therapy

## Abstract

**Background:**

Cuproptosis (copper death) is a recently found cell death type produced by copper iron; nonetheless, the properties of cuproptosis molecular subtypes and possible involvement of cuproptosis-related genes (CRGs) in the tumor microenvironment (TME) in ovarian cancer (OC) remain unknown.

**Methods:**

CRG changes were characterized at the genomic and transcriptional levels in 656 OC samples, and their expression patterns were investigated using three different datasets.

**Results:**

We identified three distinct molecular subtypes, and discovered that variations in molecular subtypes were linked to patient prognosis, TME cell infiltration characteristics, malignancy, and immune-related pathways. Then, in order to predict overall survival (OS), we created a risk score and tested its predictive potential in OC patients. As a result, we created a very accurate nomogram to increase risk score clinical applicability. Better OS, younger age, early stage, and immune activity were all associated with a low risk score. The hallmarks of a high-risk score are older age, advanced stage, immunosuppression, and a bad prognosis. Furthermore, risk score was linked to immune checkpoint expression (including PD-L1, CTLA4), targeted therapy gene expression (PARP, PDGFRA), cancer stem cell (CSC), chemotherapy and targeted medication sensitivity.

**Conclusions:**

Our comprehensive analysis of CRGs in OC showed their potential role in TME, clinicopathological characteristics, chemotherapy and targeted drug screening and prognosis. These discoveries could help us better understand CRGs in OC, as well as pave the path for novel ways to assess prognosis and design more effective immunotherapy strategies.

**Supplementary Information:**

The online version contains supplementary material available at 10.1186/s12935-022-02756-y.

## Introduction

Ovarian cancer (OC) is the most lethal malignant gynecological tumor. Owing to the lack of early clinical symptoms and sensitive and specific diagnostic markers, more than 70% of the patients were found to be in an advanced stage [1]. Although most patients achieve complete remission after tumor reduction surgery and adjuvant chemotherapy, 70–80% of patients experience tumor recurrence and chemotherapy resistance [2]. Current clinical trials evaluate combinations of targeted therapies, such as antiangiogenic agents, PARP inhibitors, and immunotherapy for patients with relapse and drug resistance. In addition to different OC subtypes showing different sensitivities and resistance to treatment, studies have shown that patients with OC have considerable molecular heterogeneity at the genomic and immunological levels, providing a more complex landscape for the response to treatment and the local tumor microenvironment (TME) in OC [2]. Therefore, there is an urgent need for an effective classification and signature in ovarian cancer regarding targeted therapy and immunotherapy to indicate prognosis and guide clinical treatment.

Copper is an essential nutrient whose oxidation–reduction (redox) properties promote copper-dependent cell growth and proliferation (cuproplasia) and play a role in mitochondria-dependent cytotoxicity (cuproptosis) when the Cu concentration exceeds a certain threshold [3]. Many studies have shown that in various cancers, including gynecological cancer, Cu concentration in the tumors or sera of animal models and patients with cancer is increased [4], and studies have demonstrated that abnormal Cu accumulation may promote the possibility of malignant transformation through unknown mechanisms [5]. Excessive copper accumulation in the body endangers life. Many studies have confirmed that excessive copper accumulation can induce “apoptosis” [6], and copper ion carriers such as disulfiram [7] and elesclomol [8] have been used as cancer therapeutic drugs to induce copper death. However, the specific mechanism of excessive copper-induced cell death was not clarified until March 2022. Tsvetkov et al. showed that copper death is a process in which copper directly binds to lipoylated components of the tricarboxylic acid (TCA) cycle, resulting in the aggregation of the lipoylated protein and subsequent loss of the iron-sulfur cluster protein, leading to proteotoxic stress and ultimately cell death [9]. They demonstrated that the mechanism of copper-induced cell death differs from all other known regulatory cell death mechanisms, including apoptosis, ferroptosis, pyroptosis, and necroptosis. Therefore, Tsvetkov et al. proposed that this previously uncharacterized cell death mechanism be termed cuproptosis [9]. The properties of cuproptosis molecular subtypes and the potential role of cuproptosis-related genes (CRGs) in the TME in OC remain unknown. With a clear definition of cuproptosis, follow-up research based on cuproptosis-related regulatory factors in cancer will provide potential mechanisms for the occurrence and treatment of cancer and new ideas for the classification, prognosis, and prediction of treatment responsiveness of cancer.

In this study, 656 OC samples were stratified into three cuproptosis-related subtypes according to the expression levels of thirteen CRGs, and the survival and immune infiltration differences among the subtypes were explored. The patients were then divided into two gene subtypes based on the differentially expressed genes (DEGs) identified in the three cuproptosis subtypes. We further established a risk score model to predict overall survival (OS) and characterize the immune landscape of OC, which accurately predicted patient outcomes and significantly correlated with immune infiltration and the sensitivity of a variety of targeted drugs.

## Materials and methods

### OC data sets and preprocessing

From the public databases Gene Expression Omnibus (GEO) and TCGA, open OC gene expression datasets with complete clinical information annotation were downloaded. Patients who lacked information on their prognosis were omitted from the study. Three datasets were gathered, including two GEO (GSE53963, GSE73614) and one TCGA-OV (Ovarian serous cystadenocarcinoma). We used GEO (https://www.ncbi.nlm.nih.gov/geo/) to get the raw "CELL" file and then adjusted the backdrop and normalized the quantiles. The batch effect of the merged dataset is then removed using SVA’s R package. The TCGA database (https://portal.gdc.cancer.gov/) was used to gather transcriptome data (FPKM value), clinical information, and mutation information for 375 OC patients. Due to the lack of normal ovarian tissue data in the TCGA cohort, we also considered Genotype-Tissue Expression (GTEx, https://www.gtexportal.org/home/datasets) data from 88 normal ovarian samples to identify the DEGs between normal and tumor tissues. The expression data in both datasets were normalized to FPKM values and removed batch effect before comparison. Following then, a total of 656 OC patients were included in the studies. Additional file 2: Table S1 contains detailed information on the 656 patients with OC. Age, stage, grade, overall survival time, and survival status were among the clinical factors.

### Unsupervised clustering for cuproptosis-related genes

Thirteen CRGs were discovered in the prior investigations [9–12], and the complete list of these genes may be found in Additional file 2: Table S2. For consensus unsupervised clustering analysis, the "ConsensusClusterPlus" R package was used to divide all 656 OC patients into unique molecular subgroups based on CRG expression [13]. The following criteria were used to cluster the data: The cumulative distribution function (CDF) curve grew gently and smoothly at first; second, there were no small sample sizes in any of the groups. Finally, clustering boosted intra-group correlation while decreasing inter-group correlation. The rather extensive clinical information dataset GSE32062 was utilized for unsupervised clustering analysis to test the correctness of the clustering.

### Functional and pathway enrichment analysis

The Kaplan–Meier curve generated by the "survival" and "survminer" R package was used to analyze the variation in OS among different subtypes. Gene set variation analysis (GSVA) was used with the hallmark gene set (c2. cp.kegg.v7.2) produced from the MSigDB database to study changes in CRGs in biological processes [14]. The CRGs were functionally annotated using the “clusterProfiler” R package. Significant variations in gene ontology were defined as P values less than 0.05.

### Evaluation of tumor microenvironment cells in patients with OC

The single sample gene set enrichment analysis (ssGSEA) algorithm was used to assess the scores of TME cells in each OC sample [15]. The immunological and stromal scores of each patient were assessed using the ESTIMATE method [16]. All OC sample's fractions of 22 human immune cell types were calculated using the CIBERSORT algorithm. We also explored the relationships between programmed cell death 1 (PD-1), CD274 (PD-L1), cytotoxic T-lymphocyte associated protein 4 (CTLA4), poly(ADP-ribose) polymerase 1 (PARP1), PARP2, toll like receptor 8.

(TLR8), transforming growth factor beta 2 (TGFB2), and vascular endothelial growth factor A (BEGFA) expression and cuproptosis-related subtypes.

### Differentially expressed genes identification and functional annotation

The DEGs among the cuproptosis clusters were identified using the “limma” package in R with a fold-change of 0.2 and an adjusted p-value of < 0.05. Functional enrichment analysis was performed on the DEGs using the "clusterprofiler" package in R to further examine the probable activities of cuproptosis pattern-associated DEGs and find related gene functions and enriched pathways.

### Construction of the cuproptosis-related prognostic risk score

To begin, DEGs were subjected to univariate Cox regression analysis to discover the OS-related DEGs. Second, the patients were divided into separate subtype groups using an unsupervised clustering algorithm based on prognostic relevant CRG expression. Finally, all OC patients were randomly assigned to a training group (n = 328) and a test group (n = 328) in a 1:1 ratio, and the risk score was calculated using the training group and testing group. Briefly, using the "glmnet" R package, the LASSO Cox regression technique was employed to minimize the risk of over-fitting based on CRG prognostic genes [17]. We employed 10 × cross validation after analyzing the change trajectory of each independent variable to establish the risk signature. In the training group, multivariate Cox analysis was utilized to select candidate genes for establishing the prognostic risk score. Finally, the cuproptosis gene signature, termed as the risk score, was constructed using thirteen genes and their correlative coefficients acquired in the training group. The following formula was used to determine the risk score: Risk Score = $\sum (\mathrm{Expression of each gene }*\mathrm{ coefficients})$. The patients in the training group were separated into low-risk and high-risk groups based on their median risk scores, and then Kaplan–Meier survival analysis was performed. Similarly, the testing and total groups were sorted into low- and high-risk groups, with Kaplan–Meier survival analysis and the development of receiver operating characteristic (ROC) curves used to assess the signature’s predictive potential.

### Analyses of the prognostic risk score’s clinical correlation and stratification

The associations between the risk score and clinical variables (age, stage, grade) were compared. We ran univariate and multivariate analyses on the training, testing, and total groups to evaluate if risk scores were independent of other clinicopathological variables. In addition, we conducted a stratified analysis to explore if the risk score preserved its predictive capacity in different age, stage, and grade subgroups.

### Establishment and validation of a nomogram scoring system

Based on the results of the independent prognosis study, the clinical parameters and risk score were utilized to build a predictive nomogram using the "rms" package. Each variable was assigned a score in the nomogram scoring method, and the overall score was calculated by summing the scores from all variables in each sample. The nomogram was evaluated using time-dependent ROC curves for 3-, 5-, and 10-year survivals. The predictive value between the projected 3-, 5-, and 10-year survival events and the virtually observed outcomes was depicted using nomogram calibration plots.

### Verification of prognostic protein expression

The UALCAN website (http://ualcan.path.uab.edu/cgi-bin/ualcan-res.pl) is the Clinical Proteomic Tumor Analysis Consortium (CTPAC) database data mining platform that provides the proteins expression level of OC patients. Human Protein Atlas (HPA) provides the protein level of prognostic proteins in tumor and normal tissues. For immunohistochemistry (IHC) study, 50 formalin-fixed paraffin-embedded (FFPE) ovarian tissue samples (including 42 cases of ovarian serous adenocarcinoma tissues and 8 normal ovary tissues) were obtained from the pathology department of the Shengjing Hospital of China Medical University. Antibodies against SLC31A1 (ab133385, abcam, 1:200), and GTSE1 (ab272670, abcam,1:160) were used in this study. The experimental methods and evaluation criteria were as described in previous study [18], +++ staining was defined as high positive. Each tissue section was reviewed independently by two researchers to eliminate scoring error. This study was approved by the Ethics Committee of Shengjing Hospital of China Medical University. The need for written informed consent was waived due to the retrospective nature of this study.

### Assessment of immune status, cancer stem cell (CSC) index, and immunotherapy response

CIBERSORT was utilized to quantify the quantity of 22 infiltrating immune cells in diverse samples in the low- and high-risk groups to assess the proportions of immune infiltration cells in the TME. We explored the relationships between the fractions of 22 infiltrating immune cells and the risk score's thirteen genes. We also utilized boxplots to evaluate the differences in immunological check-point expression levels between the low- and high-risk groups, which were retrieved from earlier literature. We also analyzed the connections between the two risk groups and CSC. The Cancer Immunome Atlas (TCIA) web tool provides the results of comprehensive immunogenomic analyses. Tumor immunogenicity was quantitatively scored from 0 to 10 and was named the immunophenoscore (IPS). The IPS could be applied to predict the response to immune checkpoint inhibitors [19].

### Assessment of sensitivity of chemotherapy and molecular drugs

The "pRRophetic" package was used to calculate the risk signature in predicting the response to chemotherapy and molecular drugs [20]; the tissue subtype was limited to “ovary”, and the half-maximal inhibitory concentration (IC50) was calculated using ridge regression between the low- and high-risk groups among 251 common chemotherapeutic agents.

### Statistical analysis

Statistical analyses were performed using R software (version 4.1.0) and RStudio (version 2021.09.1 Build 372 for macOS). The Mann–Whitney U test was used to compare the differences between two groups. All tests were two sided, and a p < 0.05 was considered statistically significant unless stated otherwise, the asterisk in figures represents the p value (*p < 0.05; **p < 0.01; ***p < 0.001).

## Results

### Genetic and transcriptional change of CRGs in OC

**Fig. 1:**
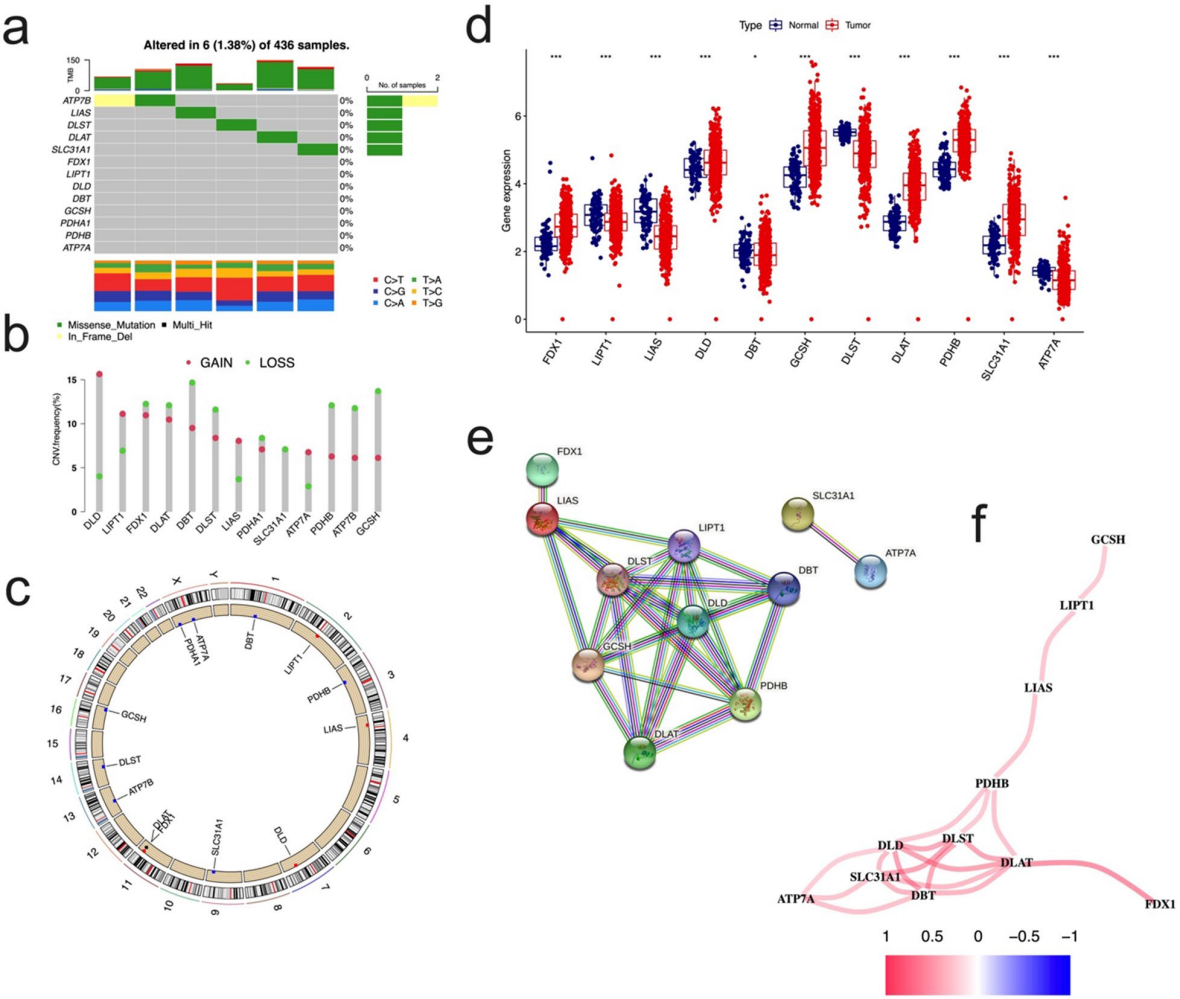
Genetic and transcriptional alterations of CRGs in OC. **a** Mutation frequencies of 13 CRGs in 436 OC patients from the TCGA cohort. **b** Frequencies of CNV gain, loss, and non-CNV among CRGs. **c** Locations of CNV alterations in CRGs on 23 chromosomes. **d** Expression distributions of 11 differentially expressed CRGs between normal ovary and OC tissues. **e** PPI network showing the interactions of the CRGs (interaction score = 0.9). **f** The correlation network of the CRGs (red line: positive correlation; blue line: negative correlation. The depth of the colours reflects the strength of the relevance). CRGs, cuproptosis-related genes; OC, ovarian cancer; TCGA, The Cancer Genome Atlas; CNV, copy number variant

Our summary analysis of the incidence of somatic mutations in 13 CRGs showed that 6 (1.338%) of 436 OC samples of TCGA had CRG mutations (Fig. 1a). Among them, the mutation frequency of ATP7B was the highest, followed by LIAS, DLST, DLAT and SLC31A1, while the other 8 CRGs (FDX1, LIPT1, DLD, DBT, GCSH, PDHA1, PDHB and ATP7A) had no mutation. Next, we studied somatic copy number changes in these CRGs and found common copy number changes in all 13 CRGs. Among them, DLD, LIPT1, LIAS had extensive increase in copy number variation (CNV), while DBT, PDHB, ATP7B, and GCSH showed decrease in CNV (Fig. 1b). The location of CNV changes in CRG on their respective chromosomes is shown in Fig. 1c.

We further compared the mRNA expression levels of 13 CRGs in OC and normal ovarian tissues, and found that 11 CRGs had significant differential expression, of which 6 genes were highly expressed in OC and 5 showed low expression (Fig. 1d). Among CNV increasing genes, only DLD in ovarian cancer samples was higher than that in normal ovarian tissues, while the expression levels of DBT, PDHB, ATP7B, etc. with CNV deletion were not consistent with CNV changes. In order to further explore the interaction of these CRGs, we conducted protein–protein interaction (PPI) analysis. We found that there were extensive interactions among the other 11 CRGs except SLC31A1 and ATP7A. The minimum interaction score required for PPI analysis was set to 0.9 (highest confidence). We determined DLD was hub gene (Fig. 1e). The correlation network of 13 CRGs is shown (Fig. 1f).

### Identification of cuproptosis subtypes in OC

**Fig. 2:**
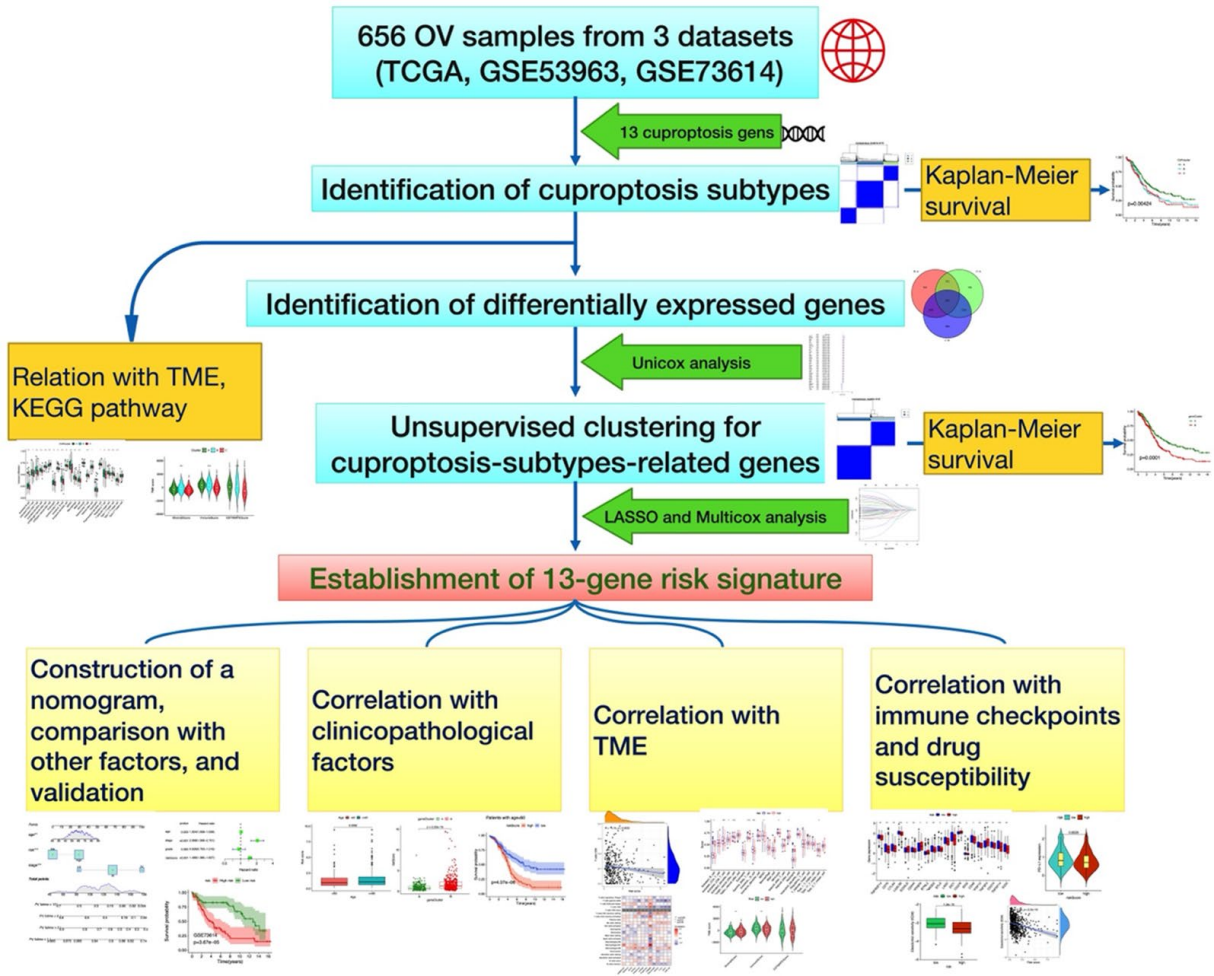
Flowchart of the entire analytical process in the study

**Fig. 3:**
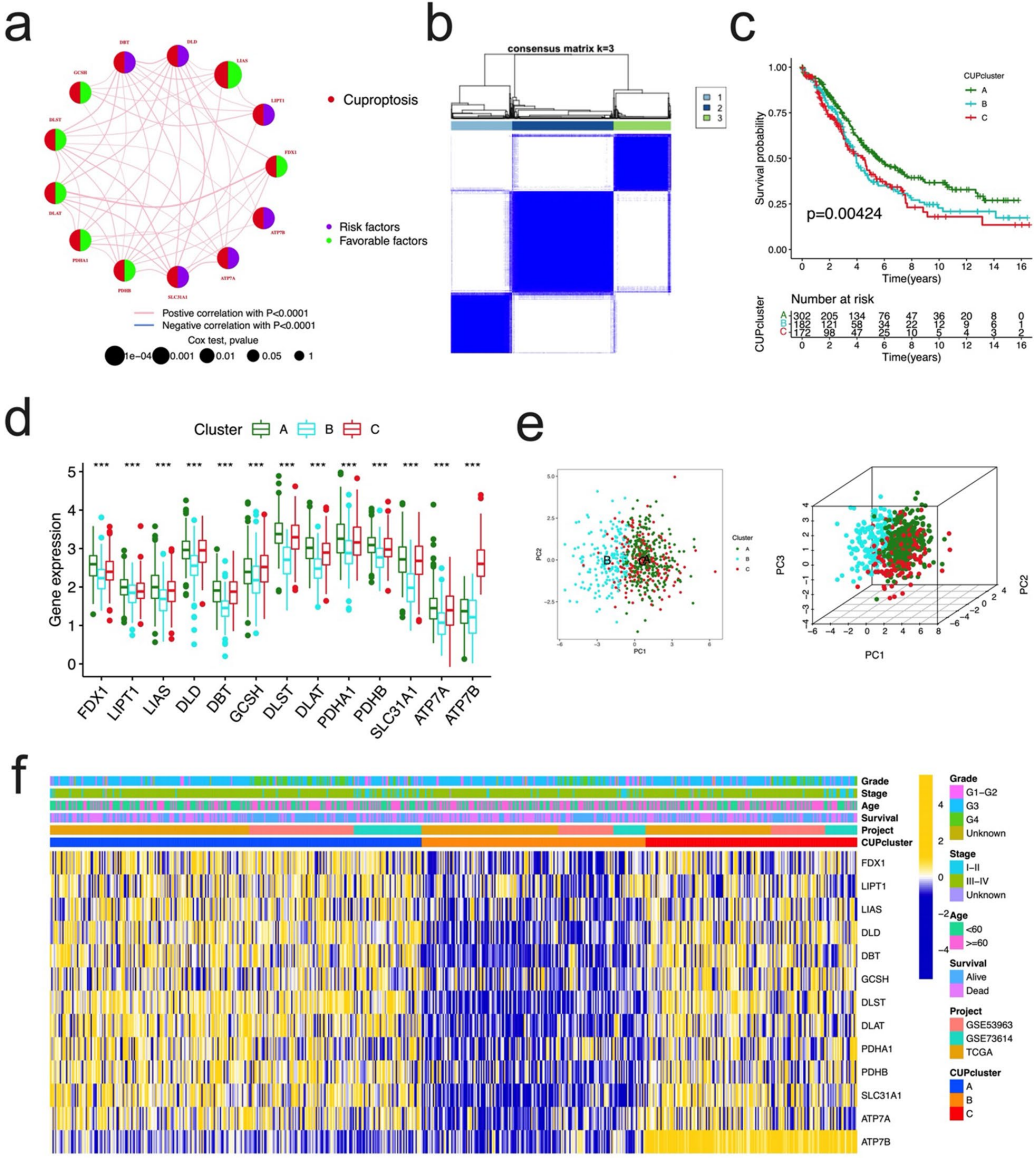
Landscape of the cuproptosis-related genes and biological characteristics of cuproptosis subtypes in ovarian cancer. **a** Interactions among CRGs in OC. The line connecting the CRGs represents their interaction, with the line thickness indicating the impact of each cuproptosis gene on the prognosis, pink lines represent positive correlations, light-blue lines represent negative correlations. The p value calculated by log-rank test. Green dots in the circle represent protective factors and violet dots represent risk factors. **b** Consensus matrix of OC patients, k = 3, using the unsupervised consensus clustering approach. **c** Kaplan–Meier curves for overall survival of all OC patients (TCGA + GSE53963 + GSE73614) cohort with three cuproptosis subtypes, the significant differences were observed among the three subtypes (log-rank test, p = 0.0003). **d** The 13 CRGs expression difference in OC patients stratified by 3 clusters (all p < 0.0001). **e** Principal component analysis of 13 CRGs in all OC cohort identified three distinct subtypes (left panel, 2 dimensions; right panel, 3 dimensions). **f** Differences in clinicopathologic features and expression levels of 13 CRGs in all OC cohorts among the three distinct subtypes. Tumor stage, age, survival status and cluster were used as patient annotations. Yellow and blue represent high and low expression of cuproptosis genes respectively

The flowchart in this study is illustrated in Fig. 2. To fully understand the expression pattern of CRG involved in ovarian tumorigenesis, 656 patients from 3 eligible OC cohorts (TCGA-OV and GSE53963, GSE73614) were integrated in our study for further analysis. Detailed information on the 656 OC patients is presented in Additional file 2: Table S1. The results of univariate Cox regression and Kaplan–Meier analysis revealed the prognostic values of 9 CRGs in patients with OC, determined the optimal cutoff value through the ‘surv_cutpoint’ function, and p < 0.05 was selected as the threshold for filtering (Additional file 1: Fig. S1, Additional file 2: Table S3). Next, we performed a multivariate Cox regression analysis on 9 prognostic CRGs, DLD and LIAS were identified as independent predictive factors (Table 1). The comprehensive landscape of 13 CRGs interactions, interconnection of the genes and their impact on the prognosis of patients with OC was constructed in a network map (Fig. 3a).

**Table 1:** Multivariate Cox regression analysis of 9 prognostic CRGs associated with overall survival in patients with OC

id	coef	HR	HR.95L	HR.95H	p value
DLD	0.37389554	1.45338532	1.10529575	1.91109835	0.0074361
LIAS	-0.3081282	0.73482111	0.57643867	0.93672076	0.01285561

To further explore the expression characteristics of 13 CRGs in OC, we used a consensus clustering algorithm to categorize the patients with OC (Additional file 1: Fig. S2). According to the clustering criteria, we chose k = 3 to be an optimal selection for sorting the entire cohort (Fig. 3b). Thus, 3 subtypes, designated Cluster A, B, and C, respectively, were identified, in which Cluster A included 302 cases, Cluster B included 182 cases, and Cluster C included 172 cases. Kaplan–Meier survival analysis revealed that OS differed among the three subtypes, and Cluster C had the worst survival preference (log-rank test, p = 0.00424, Fig. 3c). Because GSE32062 dataset has relatively complete clinical information and large sample size (n = 260), we used this dataset for external verification of the repeatability of clustering. The "ConsensusClusterPlus" R package was used for unsupervised consensus clustering, and three different subtypes were clearly identified again (Additional file 1: Fig. S3a). Moreover, survival analysis showed that there were significant differences in survival rates among the three subtypes (p = 0.0329, Additional file 1: Fig. S3b), which further demonstrated that there were three subtypes of CRGs in OC. There were significant differences in the expression of the 13 CRGs in the three clusters (Fig. 3d). Next, principal component analysis (PCA) revealed significant differences in the three subtypes (Fig. 3e). Moreover, the relationship between the three subtypes and various clinicopathological factors (survival status, age, stage, grade) was explored, the significant CRGs expression pattern was shown in different clusters, in which most of the CRGs were highly expressed in Cluster A (Fig. 3f).

### Characteristics of TME and biological function in different cuproptosis subtypes

**Fig. 4:**
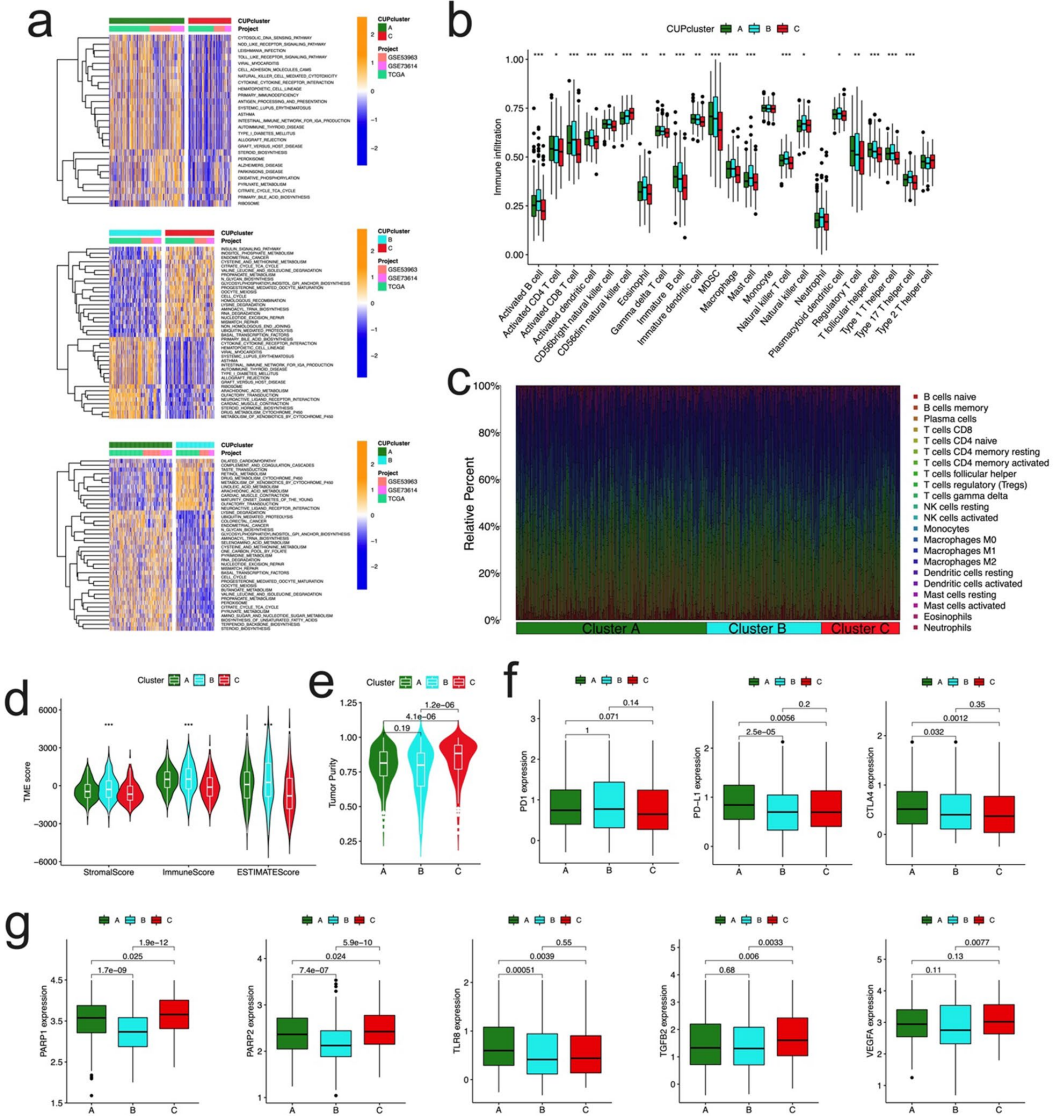
Correlations of TME and biological characteristics in three OC subtypes. **a** GSVA of KEGG biological pathways in three cuproptosis subtypes. Orange represents activation of biological pathways and blue represents inhibition of biological pathways, respectively. **b** Comparison of the ssGSEA scores for immune cells in the three OC subtypes. The line in the box represents the median value. **c** The relative percentage of subpopulations of immune cells in OC samples from total cohort stratified by three clusters. **d** Comparison between the TME score (stromal score, immune score, and Estimate score) and three OC clusters. **e** Comparison between the tumor purity and three OC clusters. **f** Expression levels difference of PDL1, PD-L1, and CTLA4 in the three OC clusters. **g** Expression levels difference of PARP1, PARP2, TLR8, TGFB2, and VEGFA in the three OC clusters. TME, tumor microenvironment; GSVA, gene set variation analysis; ssGSEA, single sample gene set enrichment analysis

To examine the functional and biological differences among three subtypes, GSVA enrichment analysis was performed (Fig. 4a, Additional file 2: Table S4). The results showed that, Cluster A was mainly enriched in some immune and carcinogenesis pathways, such as NOD/TOLL like receptor signaling pathway, natural killer cell mediated cytotoxicity, cytokine-cytokine receptor interaction, antigen processing and presentation, mismatch repair, and cell cycle, etc.; Cluster B was mainly related to immune reactions, and biological functions, such as cytokine receptor interaction, intestinal immune network, complement and coagulation cascades, drug metabolism cytochrome p450, arachidonic acid metabolism, etc.; and Cluster C was mainly enriched in carcinogenesis, and cuproptosis-related reactions, such as endometrial cancer, cell cycle, mismatch repair, inositol phosphate metabolism, citrate cycle TCA cycle, propanoate metabolism, etc. We noticed that there were some common pathways enriched both in Cluster A and C, such as glycosylphosphatidylinositol GPI anchor biosynthesis, propanoate metabolism, citrate cycle TCA cycle, etc. Considering the huge difference in prognosis between the clusters, these results suggested that CRGs are involved in immune response and cancer progression in ovarian cancer, which has a great impact on the prognosis of patients. We further compared the enrichment score of immune cells in the three subtypes by employing the ssGSEA analysis (Fig. 4b). In Cluster A, the most significant immune-infiltrating cells were activated CD4 T cell, immature dendritic cell, regulatory T cell; while the Activated B cell, CD8 T cell, dendritic cell, as well as CD56b right natural killer cell, eosinophil, immature B cell, MDSC, macrophage, mast cell, and natural killer cell showed the most infiltration in Cluster B; however, in Cluster C, only the CD56dim natural killer cell was enriched.

To further explore the differences in the composition of TME-infiltrating cells among the three clusters, the relative percentage of the 22 kinds of immune cells in each patient was calculated using the CIBERSORT algorithm (Fig. 4c). We also evaluated the TME score (stromal score, immune score, and estimate score) of the three subtypes using the ESTIMATE package, the results demonstrated the lowest TME scores in patients with Cluster C (Fig. 4d). As to tumor purity, the Cluster C has the highest score compared to Cluster A and B (Fig. 4e). Clinically, the research and development of immune and targeted therapy for malignant tumors is in the ascendant. We explored the relationship between the three clusters and gene expression of common immune therapy and targeted therapy genes, we noticed that although the PD1 expression showed no significant differences in three clusters, the expression of PD-L1 and CTLA4 demonstrated significantly difference among three clusters, whereas the Cluster A was highest than the other two groups, suggesting these OC patients would attain potential benefits in target therapy (Fig. 4f). What’s more, the expression of PARP1, PARP2, TLR8, TGFB2, and VEGFA showed significantly differences among three clusters (Fig. 4g).

### Identification of gene subtypes based on DEGs

**Fig. 5:**
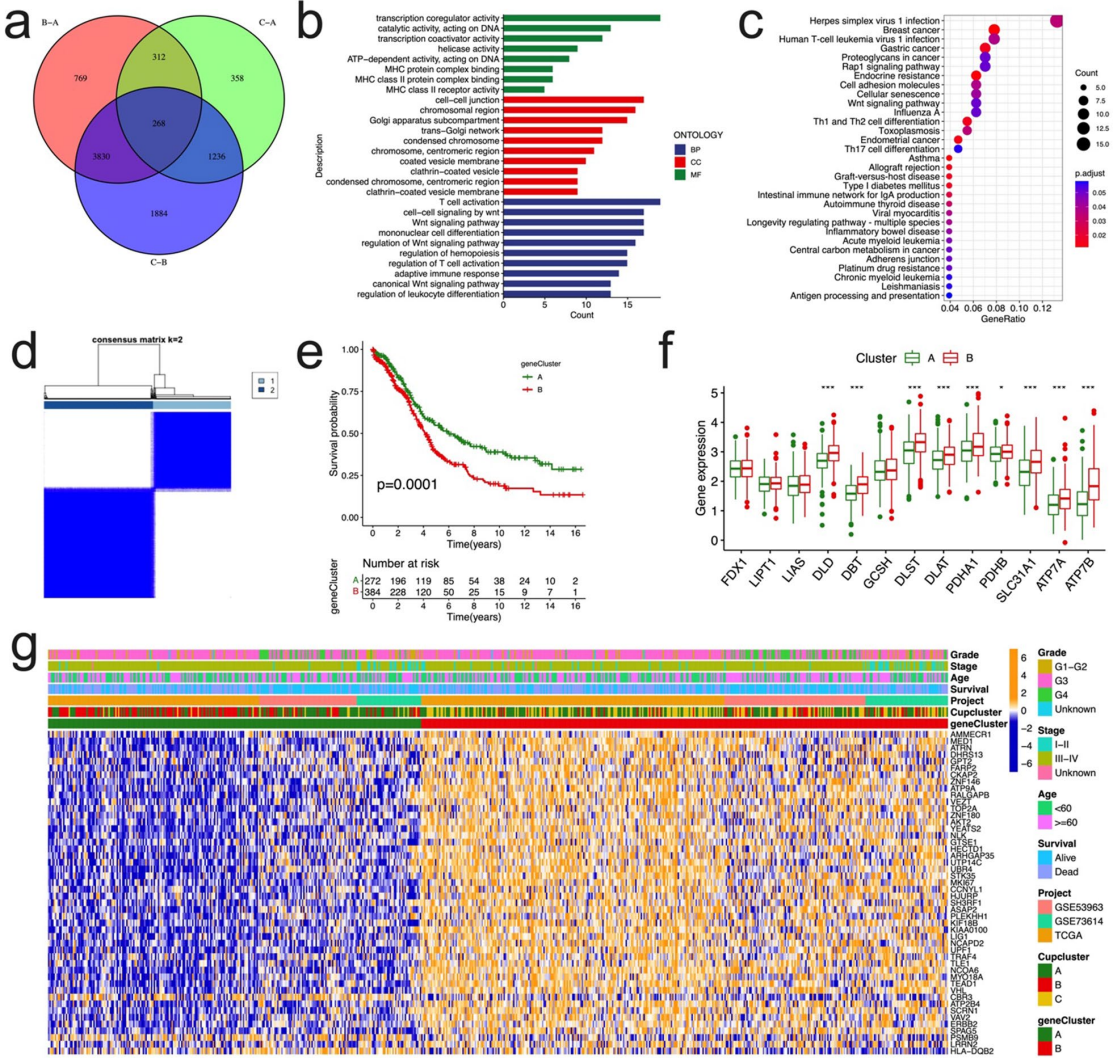
Identification of gene subtypes based on the DEGs of cuproptosis-related clusters. **a** Venn diagram to identify the DEGs among three subtypes in OC. **b** GO enrichment analysis of DEGs. The bar length represents the enriched gene counts. BP, biological process; CC, cellular component; MF, molecular function. **c** KEGG enrichment analysis of DEGs. **d** Identification of gene subtypes based on prognostic DEGs among three cuproptosis subtypes in OC cohort. **e** Kaplan–Meier curves for overall survival of all OC patients with two gene subtypes (log-rank test, p = 0.0001). **f** Differences in the expression of 13 CRGs among the two gene clusters. **g** Heatmap showing the relationships between clinicopathologic features and the two gene subtypes. DEGs, differentially expressed protein-coding genes; GO, Gene Ontology; CRGs, cuproptosis-related genes

In order to explore the potential biological function of the each cuproptosis subtype in OC, the “limma” R package was used to identify the DEGs among the three clusters, and 268 cuproptosis subtype-related DEGs were obtained (Fig. 5a). Gene ontology enrichment analysis showed the DEGs that were considerably enriched T cell activation, Wnt signaling, and adaptive immune response, etc. (Fig. 5b, Additional file 2: Table S5). KEGG analysis indicated that multiple immune and tumor-related pathways were enriched (Fig. 5c, Additional file 2: Table S6). These results suggested that cuproptosis plays a vital important role in the immune regulation of TME, tumorigenesis and development of ovarian cancer.

Then we performed univariate Cox regression analysis to determine the prognostic value of 268 cuproptosis subtype-related DEGs, and screened 48 genes related to overall survival (p < 0.05), which were used for subsequent analysis (Additional file 2: Table S7, Additional file 1: Fig. S4). To further explore the regulation mechanism of the 48 prognostic genes, a consensus clustering algorithm was carried out to divide the OC patients (Additional file 1: Fig. S5). We chose k = 2 to divide all OC patients to 2 subtypes, namely gene Cluster A, and gene Cluster B (Fig. 5d), further survival analysis demonstrated that overall survival differed significantly among the two gene subtypes (log-rank test, p = 0.0001, Fig. 5e). As expected, there were significant differences in the expression of the CRGs among the two gene clusters, in which most of the CRGs were highly expressed in gene Cluster B (Fig. 5f). Moreover, the relationship between the two gene clusters and various clinicopathological factors (survival status, age, stage, grade) was explored (Fig. 5g).

### Construction and validation of the cuproptosis prognostic signature

**Fig. 6:**
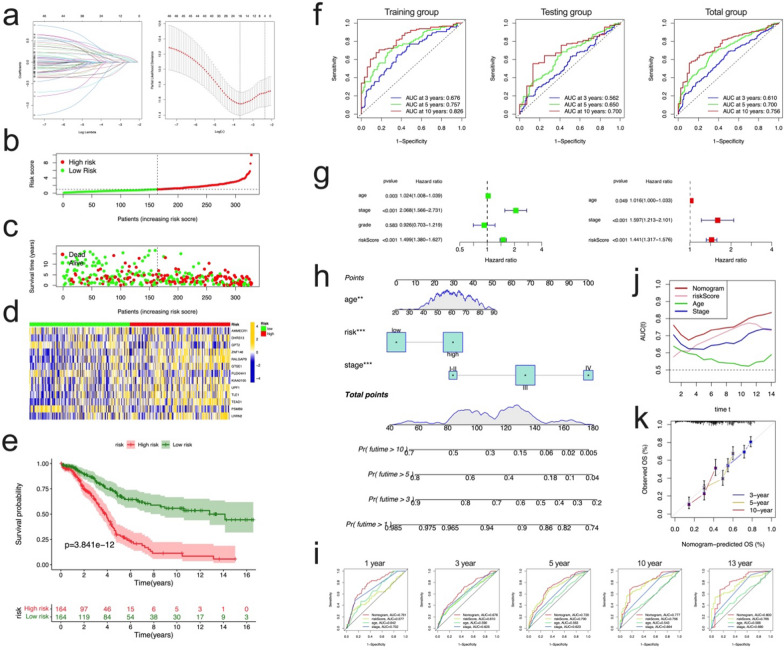
Construction of cuproptosis prognostic signature. **a** The LASSO regression analysis and partial likelihood deviance on the prognostic genes. **b** Ranked dot showing the risk score distribution and patient survival status in the training group. **c** Scatter plot showing the risk score distribution and patient survival time in the training group. **d** Heatmap showing the expression of 13 risk genes in the training group. **e** Kaplan–Meier analysis of the overall survival between the high and low risk groups in the training group. **f** AUC curves to predict the sensitivity and specificity of 3-, 5-, and 10-year survival according to the risk score in training, testing, and total groups. **g** The uniCox (left panel) and multiCox (right panel) analysis to determine the independent risk factors in the training group. **h** Nomogram for predicting 1-, 3-, 5-, and 10-year overall survival for OC patients in the total group. **i** AUC curves to predict 1-, 3-, 5-, 10-, and 13-year survival in the total group, the comparing factors includes nomogram score, risk score, age, and stage. **j** The time-dependent AUC showing the comparison of nomogram score, risk score, age, and stage in time range from 1 to 14 years in the total group. **k** Calibration curve of nomogram for predicting of 3-, 5-, and 10-year overall survival in the total group

We established a prognostic model of cuproptosis based on prognostically significant DEGs. Firstly, we randomly divided the patients into training group (n = 328) and test group (n = 328) according to the ratio of 1:1 using the “caret” package in R. LASSO analysis was applied to the 48 prognostic genes to determine the optimal value of λ, subsequent multicox analysis to further select optimal diagnostic signature. LASSO analysis suggested 16 genes remaining according to the minimum partial likelihood deviation (Fig. 6a); subsequently, we conducted multivariate Cox regression analysis on these 16 genes. We finally obtained 13 genes to construct risk models, including ZNF146, UPF1, TLE1, TEAD1, RALGAPB, PSMB9, PLEKHH1, LRRN2, KIAA0100, GTSE1, GPT2, DHRS13, AMMECR1. The correlation coefficients are provided in Additional file 2: Table S8. According to the results of multivariate Cox regression analysis, our cuproptosis risk score is constructed as follows:

Risk score = the expression of ZNF146 * 0.34 + the expression of UPF1 * 0.263 + the expression of TLE1 * 0.33 + the expression of TEAD1 * 0.345 + the expression of RALGAPB * 0.405 + the expression of PSMB9 * − *0.265* + the expression of PLEKHH1 * − *0.458* + the expression of LRRN2 * 0.251 + the expression of KIAA0100 * − *0.709* + the expression of GTSE1 * 0.252 + the expression of GPT2 * − *0.607* + the expression of DHRS13 * 0.501 + the expression of AMMECR1 * − *0.453.*

All patients were divided into a low-risk group (n = 164) and a high-risk group (n = 164) according to the median value of the risk score in the training group. In the training group, we observed that the risk distribution plot showed that survival times increased with increased risk scores (Fig. 6b, c). Furthermore, the expression of the risk genes was evidently different between the high- and low-risk groups (Fig. 6d). The survival rate was significantly lower among those with a high-risk score than among those with a low-risk score, and the Kaplan–Meier survival curve showed an apparent difference in the survival between the groups (Fig. 6e, p < 0.001). To verify the prognostic performance of the risk score, we implemented internal (testing and total groups, Additional file 1: Fig. S6) and three external GSE datasets (GSE53963, GSE73614, and GSE140082) (Additional file 1: Fig. S7–S9) validation. The risk score value was calculated according to the formula used in the training group, and the patients were divided into the low-risk or high-risk groups by the median risk score value or cutoff value calculated using the “surv_cutpoint” package in R. Heatmap indicated that the expression of 13 genes was significantly different between the low- and high-risk groups, and survival analysis revealed an evidently better prognosis in the low-risk group relative to that in the high-risk group (log-rank test; all p < 0.05, Additional file 1: Figure S7–S9).

Analysis of prognostic prediction classification efficiencies showed that the risk score had relatively high AUC values, the 5-, and 10-year AUC values in training, testing, and total groups were 0.757–0.826, 0.650–0.700, and 0.700–0.756, respectively (Fig. 6f), indicating that the risk score had excellent ability to predict the survival of OC patients. Univariate and multivariate Cox regression analysis were applied in the three groups (training group, Fig. 6g; testing group and total group, Additional file 1: Fig. S6e, f), the results showed that the risk score, as well as age, stage, were independent prognostic factors for OC.

Based on these three independent prognostic factors, a nomogram was established to predict the 1-, 3-, 5-, and 10-year OS (Fig. 6h). Compared with the risk score, age, and stage, the results of our AUC values on the nomogram model showed higher accuracy for OS at 1, 3, 5, 10, and 13 years in the total group (Fig. 6i). Time-dependent AUC plot showed the AUC value of nomogram are better than that of risk score, age, and stage in 1–14 years (Fig. 6j), suggesting that the nomogram exhibited superior survival predictive ability compared to the other factors. The subsequent calibration plots suggested that the proposed nomogram had a similar performance compared to an ideal model (Fig. 6k).

### Verification of the expression levels of prognostic proteins

**Fig. 7:**
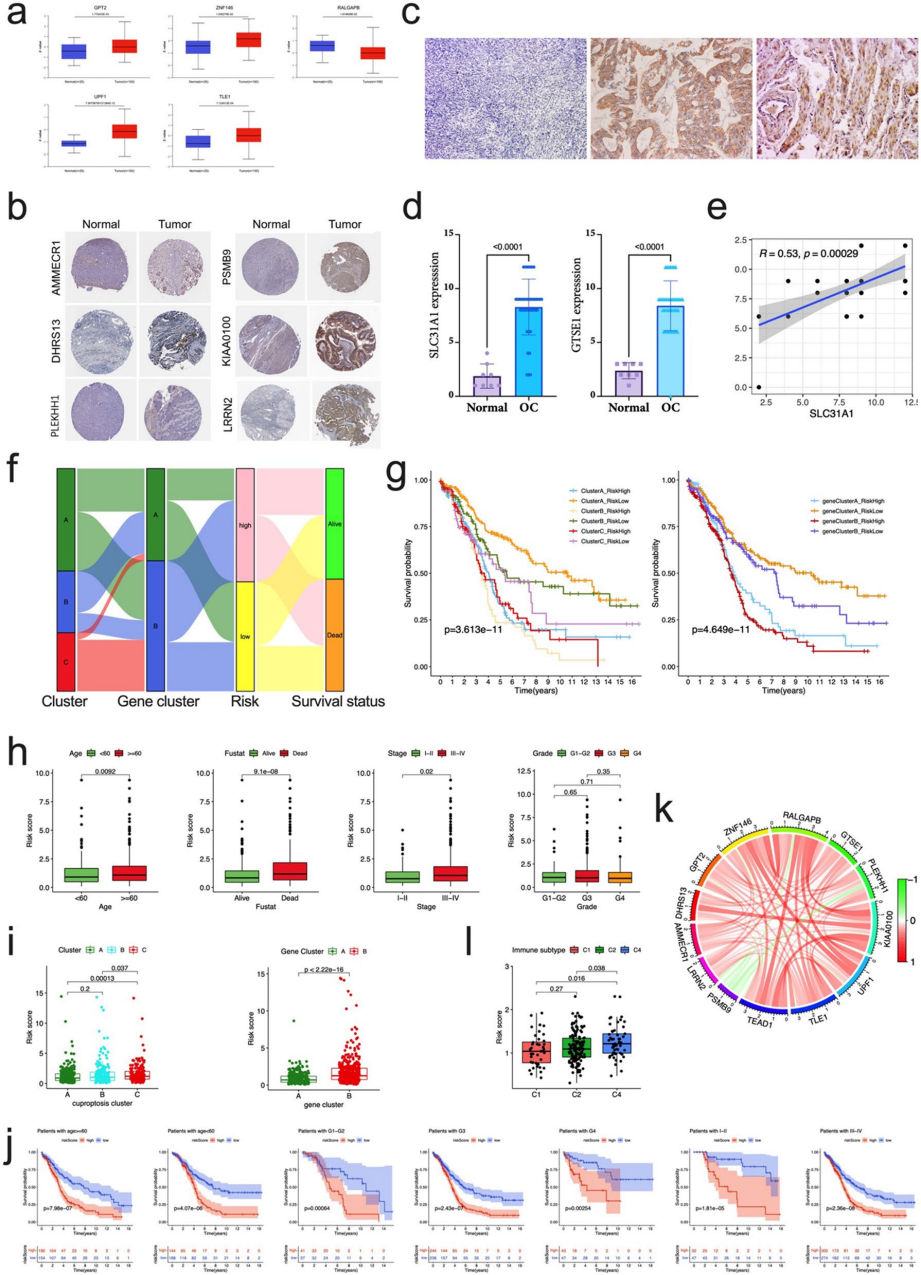
External validation for risk signature, and the correlation of risk signature and clinicopathological factors. **a** Comparison of protein expression levels of GPT2, ZNF146, RALGAPB, UPF1, and TLE1 in OC tissues and normal ovary tissues by CTPAC database. **b** Representative protein expression levels of AMMECR1, DHRS13, PLEKHH1, PSMB9, KIAA0100, and LRRN2 in OC tissues and normal ovary tissues by HPA database. **c** Typical IHC staining pictures for normal ovarian tissue (left), and positive staining of SLC31A1 (middle), and positive staining (right) for GTSE1 in OC tissues. **d** Expression difference of SLC31A1 and GTSE1 in normal and malignant ovarian tissues by IHC staining. **e** The correlation of SLC31A1 and GTSE1 staining in OC tissues. **f** An alluvial diagram of the distribution of cuproptosis cluster, gene cluster in two risk groups, as well as survival outcomes. **g** The Kaplan–Meier survival curves were stratified by cuproptosis cluster and risk subgroup (left panel), as well as gene cluster and risk subgroup (right panel). **h** The comparison of clinicopathological factors (age, stage, survival status, grade) with risk score. **i** Difference of risk score among three cuproptosis clusters (left panel) and gene clusters (right panel). **j** The Kaplan–Meier survival curves were stratified by age (< 60 years and ≥ 60 years), grade (G1–G2, G3, and G4), stage (Stage I–II, and III–IV) in two risk groups. **k** The co-relationship of 13 proteins in the prognostic signature. **l** Difference of risk score among the immune subgroups (C1-Wound Healing, C2-IFN-gamma Dominant, C4-Lymphocyte Depleted)

We analyzed the expression of prognostic proteins in OC and normal ovary tissue from the CPTAC database. The RALGAPB protein expression level was decreased in OC tissue compared with normal ovary tissue, while the expression levels of GPT2, ZNF146, UPF1, and TLE1 were significantly higher in OC tissues (Fig. 7a). In the HPA data, compared with normal tissues, DHRS13, PLEKHH1were expressed at medium to high levels in tumor tissues, while the expression of PSMB9, and LRRN2 was significantly up-regulated in OC samples, and AMMECR1, KIAA0100 slightly downregulated in OC samples (Fig. 7b). The correlation of the CRGs and risk genes were showed in Additional file 1: Fig. S10, we noticed that nearly all of them were positively correlated. We chose one CRG (SLC31A1) and one risk gene (GTSE1) for IHC staining validation. IHC staining data from 50 clinical samples in our hospital indicated that both of the expression scores of SLC31A1 and GTSE1 in ovarian malignant tumors were significantly higher than those in normal ovarian tissues (all p < 0.0001, Fig. 7c, d), they were positively correlated (R = 0.53, P < 0.001, Fig. 7e). In addition, we further analyzed the relationship between the expression of SLC31A1 and GTSE1 and the clinicopathological characteristics of OC patients. The high expression of SLC31A1 and GTSE1 was related to advanced stages (all p < 0.05, Table 2), and there were no obvious correlations of SLC31A1 and GTSE1 with age, and grade (Table 2).

**Table 2:** Relationship between SLC31A1, GTSE1expression and clinicopathological features of 42 OC cases

Variates	Low			High	High positive rate (%)	p
	–	+	+ +	+ + +		
SLC31A1						
Age						
≤ 60 y	1	2	10	11	45.80	0.179
> 60 y	1	1	4	12	66.70	
FIGO stage						
I–II	1	1	11	7	35.00	0.014
III–IV	1	2	3	16	72.70	
Grade						
Well	1	1	4	6	50	0.337
Moderate	1	1	6	6	42.90	
Poor	0	1	4	11	68.80	
GTSE1						
Age						
≤ 60 y	0	1	13	10	41.70	0.108
> 60 y	0	0	6	12	66.70	
FIGO stage						
I–II	0	0	14	6	30.00	0.006
III–IV	0	1	5	16	72.70	
Grade						
Well	0	0	7	5	41.70	0.538
Moderate	0	1	6	7	50.00	
Poor	0	0	6	10	62.50	

### Clinical correlation analysis and stratification analysis of the prognostic risk score

The alluvial diagram was used to better visualize the survival differences among the different cuproptosis clusters, gene clusters, risk score, and the survival status (Fig. 7f). The majority of patients in cuproptosis cluster A were also in the low-risk group, which had a good prognosis, similarly, gene cluster B with high-risk score patients had the worst survival (Fig. 7g). Next, the relationship between the risk score and cuproptosis clusters, cuproptosis gene clusters, as well as the clinicopathological factors, were further explored. We found that older patients (≥ 60 years), advanced stage (Stage III-IV), and death were significantly correlated with higher risk scores, whereas the grade showed no correlation with risk score (Fig. 7h). What’s more, cuproptosis Cluster C and gene Cluster B were significantly correlated with more risk scores (Fig. 7i). Further survival analysis showed that the risk scores could accurately predict the prognosis of patients with all the stratified varies of clinicopathological factors (all p < 0.01, Fig. 7j). These results suggest that the risk score may be associated with immunity and tumor-related pathways, may be helpful in predicting the cuproptosis subtype in OC, and predicting the survival of patients. The correlation of the proteins included in the prognostic signature showed that most of the proteins are positive correlated, whereas the PSMB9 displayed the strongest negative correlation (Fig. 7k).

Furthermore, based on the results of immunotyping of pancancer in the literature [21], we compared the relationship between risk score and immunotyping of ovarian cancer, and found that there were significant differences between the three existing immune subtypes C1 (wood healing), C2 (IFN gamma dominant), C4 (lymphocyte completed) and risk score in TCGA data set of ovarian cancer. The risk score in C4 group was significantly higher than that in C1 and C2 groups (Fig. 7l), suggesting that there is a potential relationship between our risk signature and immune microenvironment, survival, etc.

### Association of the prognostic signature and tumor infiltrating immune cells

**Fig. 8:**
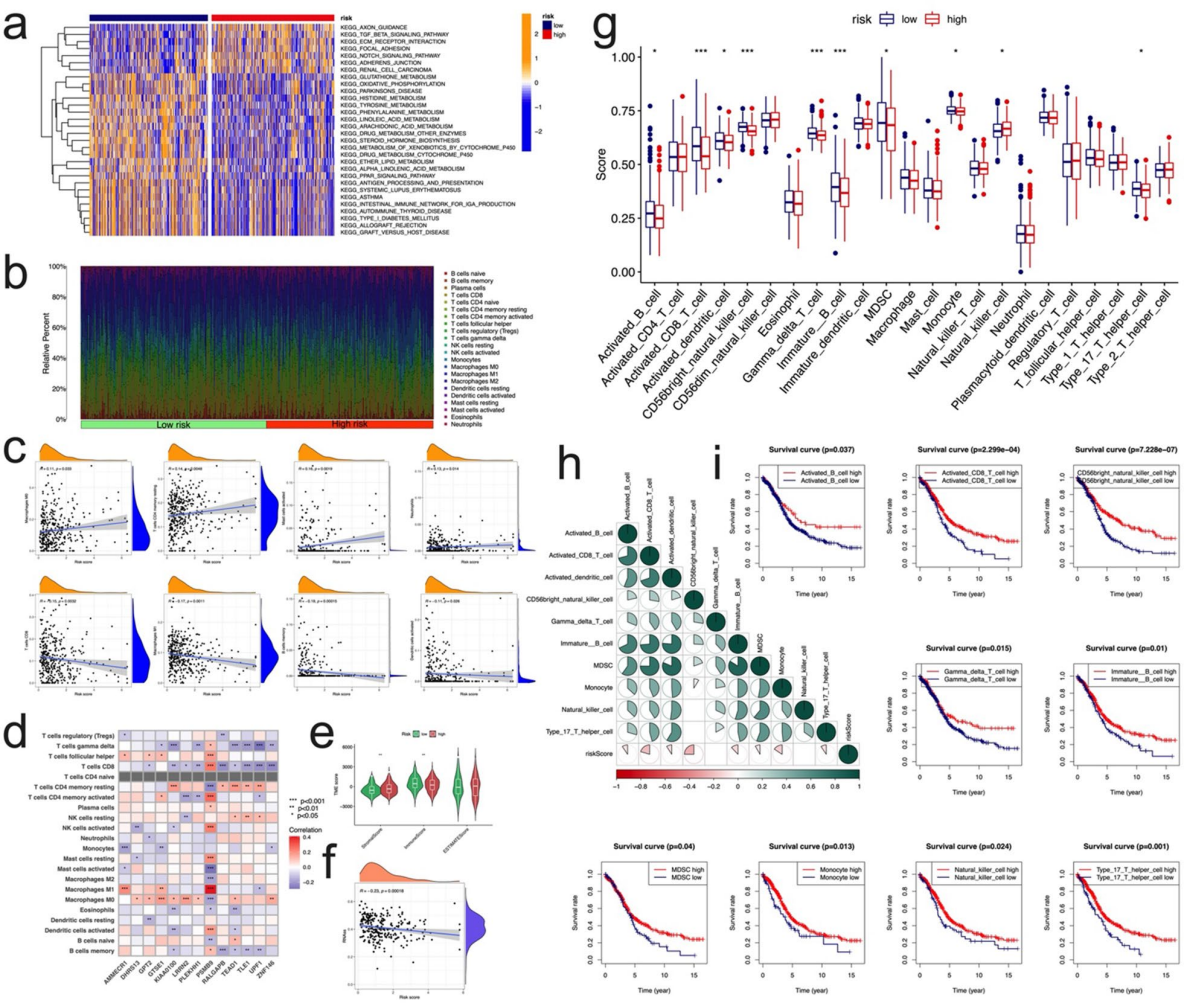
The landscape of immune microenvironment with prognostic signature. **a** GSVA of KEGG biological pathways in high and low risk groups. Orange represents activation of biological pathways and blue represents inhibition of biological pathways, respectively. **b** The relative percentage of subpopulations of immune cells stratified by high and low risk groups. **c** Correlations between risk score and immune cell type based on CIBERSORT. **d** Correlations between the abundance of immune cells and 13 genes of risk signature. **e** Correlations between risk score and immune score, stromal score, and ESTIMATE score. **f** Relationship between risk score and CSC index. **g** Comparison of the ssGSEA scores for immune cells of patients between the high- and low-risk groups. The line in the box represents the median value. **h** Correlation matrix of differentially expressed immune cells and the risk score. **i** Survival curves obtained by the Kaplan–Meier method indicated that high proportions of the 9 differentially expressed immune cells were significantly associated with prolonged overall survival

GSVA analysis showed that the immune and tumor related pathways were enriched, such as TGFbeta signal, Notch signal, antigen processing, etc. (Fig. 8a). GSEA analysis demonstrated that the immune and tumor related pathways attained the most enriched (Additional file 1: Fig. S11). The above results suggest that the model has potential correlation in tumor immune infiltration. Thus, we performed the CIBERSORT algorithm to assess the association between risk score and the abundance of immune cells (Fig. 8b). We noticed that risk score was positively correlated with Macrophages M0, T cells CD4 memory resting, Neutrophils, and Mast cells activated; the risk score was negatively correlated with T cells CD8, macrophages M1, B cell memory, and Dendritic cells activated (Fig. 8c). We also evaluated the relationship between the 13 genes in the risk signature and the abundance of immune cells. We observed that most immune cells were significantly correlated with the 13 genes (Fig. 8d). A low-risk score was closely associated with a high immune score, whereas a high-risk score was associated with a high stromal score (Fig. 8e). In addition, we explored the risk score and CSC index values, and found that the linear correlation between them was observed, risk score was negatively correlated with the CSC index (R =  − 0.23, p < 0.001, Fig. 8f), indicating that OC cells with lower risk score had more significant stem cell characteristics and lower degree of cell differentiation.

Next, the amount of immune infiltrating cells between the two risk groups in the total cohort was further evaluated. Compared with low-risk group, the high-risk group exhibited decreased activated B cell, activated CD8 T cell, activated dendritic cell, CD56 bright natural killer cell, Gamma delta T cell, Immature B cell, MDSC, monocyte, type 17 T helper cell (Fig. 8g). Correlation analyses of the above differentially expressed immune cells and the risk score further demonstrated that all these cells were negatively correlated with the risk score system, while most of these cells were positively correlated with each other (Fig. 8h). In addition, immune cells correlated with prognosis, high infiltration of most of the above differentially immune cells was correlated with better survival by Kaplan–Meier analysis (Fig. 8i). In total, these results indicated that the risk score signature might reflect the infiltration level of immune cells and TME, which are responsible for adaptive antitumor immunity.

### Predicting response to immunotherapy, targeted therapy, sensitivity of chemotherapy and molecular drugs in patients with OC

**Fig. 9:**
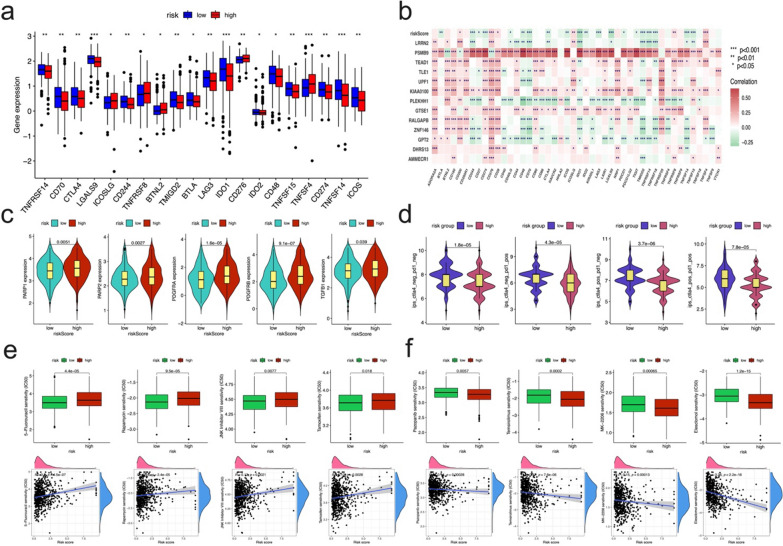
Association of the prognostic signature with immune-therapy and chemotherapy. **a** Expression of immune checkpoints between the high- and low-risk groups. **b** The correlation of immune checkpoints expressions with risk score. **c** Boxplots showed the expression difference of PARP1, PARP2, PDGFRA, PDGFRB, TGFB1, and TGFB2 between the high- and low-risk groups. **d** The relative probabilities of responding to anti-CTLA-4 and anti-PD1 antibodies in the low-risk and high-risk groups. **e** Differences in drug sensitivity between the high-risk and low-risk groups based on IC50 values of 5-Fluorouracil, rapamycin, JNK Inhibitor VIII, tamoxifen, the corresponding correlations between risk score and IC50 values were showing below. **f** Differences in drug sensitivity between the high-risk and low-risk groups based on IC50 values of pazopanib, temsirolimus, MK-2206, and elesclomol, the corresponding correlations between risk score and IC50 values were showing below

Given the importance of checkpoint inhibitor-based immunotherapies, we explored the differences in the expression of immune checkpoints between the two groups. Substantial differences were found in the expression of CD70/244/276/48/274, CTLA4, and many other important indicators between the two groups of patients (Fig. 9a). We noticed low risk group patients showed high expression of PDL1 and CTLA4, suggested they could be benefitted from PDL1 and CTLA4 immune therapy. Further correlation analysis showed that, the risk score signature was mostly negatively correlated with all these checkpoint genes, in which PSMB9 seemed to be the most positively correlated, and PLEKHH1 seemed to be the most negatively correlated (Fig. 9b).

From the perspective of practical application of clinical treatment of ovarian cancer, we evaluated the expression of common target genes of targeted drugs in ovarian cancer, we noticed the expression of PARP1, PARP2, platelet derived growth factor receptor alpha (PDGFRA), PDGFRB, and TGFB1 were significantly low expressed in low-risk group than that in high-risk group (Fig. 9c), which suggested that high-risk group patients may benefit from the corresponding targeted drugs.

In addition, we further applied TCIA to predict the susceptibility of patients to immunotherapy. We found that the low-risk group had a higher IPS than that of the high-risk group, which meant that the low-risk group may be more sensitive to immune checkpoint inhibitors (Fig. 9d). Taken together, these results indicated that the prognostic signature could predict the potential response to immunotherapy in OC patients.

Finally, we evaluated the relationship between the risk signature and the sensitivity to chemotherapy and targeted therapy drugs for OC patients by “pRRophetic” R package. Our results showed that, a significant difference was found between the two risk subgroups in the estimated IC50 values of 95 types of chemotherapy agents (all p < 0.05, Additional file 2: Table S9). The IC50 values of 5-Fluorouracil, rapamycin, JNK Inhibitor VIII, and tamoxifen were significantly lower in samples of the low-risk group than in those of the high-risk group (Fig. 9e). However, interestingly, there are still 43 drugs with low expression of IC50 in the high-risk group, the high-risk group demonstrated much higher sensitivity to the VEGFR inhibitor (pazopanib), mTOR inhibitor (temsirolimus), PI3K signal inhibitor (MK-2206) than that of the low-risk group (Fig. 9f). Among these estimated drugs, elesclomol, as an oxidative stress inducer and a highly lipophilic Cu^2+^ binding molecule, is particularly dazzling. It has become the drug with the strongest negative correlation, which means that it still has high potential application value in the targeted treatment of ovarian cancer (Fig. 9f). These results indicated that the risk score had potential predictive value for chemotherapy and targeted therapy.

## Discussion

Ovarian cancer is the deadliest gynecological cancer with a poor prognosis. This occult disease is challenging to diagnose early, relapses easily, and produces drug resistance. The clinical results of advanced OC are still unsatisfactory [22]. The difference in the molecular heterogeneity of OC provides a complex landscape for predicting the prognosis of patients and their response to immunotherapy. Therefore, the construction of a molecular subtype and characterization of the corresponding immune microenvironment can play a crucial role in improving the prognosis of patients with OC. In this study, we first analyzed the variation and expression of 13 CRGs in OC and their impact on patient prognosis. We found that most CRGs were dysregulated in OC, and DLD and LIAS were independent risk factors for the prognosis of patients with OC. Dihydrolipoamide dehydrogenase (DLD) is a mitochondrial enzyme that exhibits myocardial xanthase activity. In OC, studies have shown that it can be used as a tumor-associated antigen (TAA) to activate the immune system and produce specific autoantibodies during tumor occurrence and progression [23]. It can also be used as a new diagnostic marker of OC and produces ROS related to its redox activity [24], which plays a role in inducing the death of tumor cells. Our results showed that its expression was significantly upregulated in OC and significantly correlated with poor patient prognosis. As a mitochondrial enzyme, lipoyl synthase (LIAS) cooperates with other factors such as DLD to catalyze the final step of lipoic acid biosynthesis [25]. In this study, the expression of LIAS in ovarian cancer was downregulated significantly, and the prognostic analysis suggested that LIAS is a protective gene.

Based on the expression levels of these CRGs, we clustered OC samples into three different molecular subtypes. Patients with different subtypes had significantly different prognoses, among which those in cluster C had the worst prognosis. After exploring the reasons for these differences, our GSVA showed that clusters A and B were mainly enriched in some immune activation pathways, and cluster C was mainly enriched in carcinogenesis pathways. We can conclude that cuproptosis is closely related to tumors and immunity. Therefore, we further studied the correlation between the three subtypes and TME cell infiltration. Notably, in samples of Clusters A and B, the infiltration of immune cells such as activated B cells, activated CD4 + T cells, activated CD8 + T cells, dendritic cells, immune B cells, MDSCs, macrophages, natural killer cells, and regulatory T cells was significantly higher than that of Cluster C. Overall, the low infiltration levels of these immune cells in cluster C partly explain the poor prognosis of patients with OC. In addition, our patients with OC with different prognoses and immune infiltration were better distinguished by the three cuproptosis subtypes. They have a particular clinical application value, and they show that cuproptosis has a potential correlation with the formation of the immune microenvironment of OC. Pathway analysis of clinically significant DEGs suggested that these genes were related to tumors and immunity. Further studies have shown that these DEGs can be divided into two gene subtypes. These findings can help us understand the relationship among cuproptosis, TME cell infiltration, and OC.

Furthermore, we constructed a risk model based on 13 key genes related to cuproptosis. It can effectively distinguish the prognosis of patients and is related to age, tumor stage, survival status, cuproptosis cluster, and gene cluster. The higher the risk score, the worse the prognosis of patients, and it is an independent risk factor for the prognosis of patients with OC, verified in TCGA cohort. Furthermore, the higher the risk score, the lower the immune score, the more immune-related pathways are enriched in patients with low-risk scores, and the fewer immune inflammatory cells are activated. Specifically, the risk score is negatively correlated with immune cell infiltration, suggesting that patients in the high-risk group have immunosuppression, which further explains the previous cuproptosis clusters.

Some immune cells in the two risk groups were found to be different. While the high-risk group had more macrophages M0, T cells CD4 memory resting, neutrophils, and mast cells activated, the low-risk group had more T cells CD8, macrophages M1, B cell memory, and dendritic cells activated. The ssGSEA algorithm results also revealed that patients in the low-risk group have higher immune activity. A large number of studies have shown that dense T cell infiltration, particularly cytotoxic CD8 T cells, indicates a favorable prognosis [26, 27]; this finding has also been confirmed in ovarian cancer [28]. Macrophages play a complex role in tumor immunotherapy [29]; in most tumors, M2 macrophages are a major subtype of macrophages that have been proven to be associated with chronic inflammation and conducive to the development of tumor growth and invasive phenotype, these cells are associated with the poor prognosis of ovarian cancer, gastric cancer, and prostate cancer [26, 30]; in contrast, high-density M1 macrophages may be associated with acute inflammation and implies a good prognosis in patients with ovarian or gastric cancer [26, 30]. Traditional type 1 dendritic cells are required to elicit anti-tumor T cell responses, implying that migrated cDC1 can transmit tumor antigens and cross present to CD8 + T cells [31]. Our findings support the preceding conclusions. T-follicular helper cells are significantly correlated with high expression of PD-L1, which promotes tumor immune response [32], and CD4 T cells play a negative role in tumor immunity [33]. However, the precise role of other immune cells in OC, such as B cells and NK cells, and their impact on patient prognosis, remain unknown or debatable [28].

With an in-depth study of tumor immunology and molecular biology, immunotherapy has provided a new perspective on tumor treatment. Currently, OC immunotherapy can be divided into three categories: immune modulators, including immune checkpoint inhibitors (ICIs), cancer vaccines, targeted antibodies, and adaptive cell therapy [34]. In OC, research on ICIs targeting CTLA-4, PD-1, and PD-L1 is increasing, and clinical studies have preliminarily shown their safety and effectiveness. The effect of CTLA-4 antibody is still under study, while the effect of PD-L1 inhibitor in OC has been confirmed [35]. Several commonly used PD-1/PD-L1 inhibitors are in clinical research on OC [36], including combined application with antiangiogenic drugs [37] or the targeted drug PARP inhibitor [38]. However, in general, PD-1/PD-L1 inhibitors alone are only effective in a small number of patients with OC, and the clinical effect of a combined application is better than that of a single application, especially when combined with targeted drug PARP inhibitors. Currently, relevant research is ongoing. Therefore, immune and targeted treatments for specific types of OC through molecular typing are expected to improve the prognosis of patients effectively. In this study, cuproptosis Cluster C was in an immunosuppressive state, and the expression of PDL1 and CTLA-4 was also significantly lower than that of cluster A, suggesting that these patients with worse prognoses could not benefit from PDL1 and CTLA-4 antibody treatment. However, the expression of PARP1, PARP2, and TGFB2 in cluster C patients was significantly higher than that in the patients from the other two groups, suggesting that PARP inhibitors and gemogenovatucel-T (Vigil) are expected to be beneficial to this group of patients. In the risk model, the prognosis of high-risk patients was poor, and the expression of most immune checkpoints in the high-risk group was also downregulated, meaning these patients with poor prognoses cannot benefit from ICIs. However, the expression of PARP and PDGFR in the high-risk group was still higher than that in the low-risk group, suggesting that olaparib and pazopanib have potential and due value, which provides a useful reference for the more strategic selection of immune and targeted therapies for these patients with poor prognoses. Using the risk score, we can predict the effective chemotherapy or targeted drugs for OC, providing a good reference for the personalized treatment of patients with molecular subtypes based on cuproptosis.

Our study has a few limitations. First, all our analyses were based on data from public databases, and all the samples we used were obtained retrospectively. Thus, the results may be influenced by an inherent case selection bias. To confirm the stability of our findings, we need to conduct more extensive prospective investigations and more in vitro and in vivo experimental research. Furthermore, due to the problem of retrospective research, the data of some critical clinical variables (such as presence/absence of ascites before surgery, the levels of tumor markers, use of radiotherapy and chemotherapy, and whether the operation reached R0) cannot be used for analysis in most datasets, which may affect the immune response and prognosis evaluation of cuproptosis.

## Conclusions

Finally, our study uncovered the complex regulatory mechanism of cuproptosis in OC through which it influences the TME, clinicopathological characteristics, and prognosis. In addition, the involvement of CRGs in immune and targeted therapies has also been determined. These findings highlight the clinical importance of CRGs and offer new perspectives on how to guide tailored immunotherapy treatments for patients with OC.

## Supplementary Information

**Additional file 1:** **Figure S1.** The Kaplan-Meier plots of 9 prognostic CRGs in ovarian cancer. **Figure S2.** Unsupervised clustering of cuproptosis-related genes and Consensus matrix heatmaps for k = 2,4-9, as well as the consensus cumulative distribution function (CDF) curve for 2-9 curves. **Figure S3.** External validation for unsupervised clustering of cuproptosis-related genes in GSE32062. a Unsupervised clustering of cuproptosis-related genes in GSE32062, showing the consensus matrix heatmaps for k = 2-5; b Kaplan–Meier curves for overall survival of 260 OC patients (GSE32062) with three cuproptosis subtypes, the significant differences were observed among the three subtypes (log-rank test, p =0.0329). **Figure S4.** Forest plot of 48 prognostic genes after uniCox analysis of the DEGs (all P<0.05). **Figure S5.** Unsupervised clustering of 48 prognostic DEGs and Consensus matrix heatmaps for k = 3–9, as well as the consensus cumulative distribution function (CDF) curve for 2–9 curves. **Figure S6.** Validation of risk score in testing and total group. a Ranked dot plot indicating the risk score distribution and the ovarian cancer patients’ survival status. b Scatter dot plot indicating the risk score distribution and the ovarian cancer patients’ survival time and status. c Heatmap showing the expression distribution of 13 risk genes in high and low risk groups. d KM analysis of the overall survival between the high and low risk groups. e The unicox analysis for identifying the independent risk factors. f The multicox analysis for identifying the independent risk factors. **Figure S7.** Validation of risk score in GSE53963 dataset. a Ranked dot plot indicating the risk score distribution and the ovarian cancer patients’ survival status. b Scatter dot plot indicating the risk score distribution and the ovarian cancer patients’ survival time and status. c Heatmap showing the expression distribution of 13 risk genes in high and low risk groups. d KM analysis of the overall survival between the high and low risk groups. **Figure S8.** Validation of risk score in GSE74614 dataset. a Ranked dot plot indicating the risk score distribution and the ovarian cancer patients’ survival status. b Scatter dot plot indicating the risk score distribution and the ovarian cancer patients’ survival time and status. c Heatmap showing the expression distribution of 13 risk genes in high and low risk groups. d KM analysis of the overall survival between the high and low risk groups. **Figure S9.** Validation of risk score in GSE140082 dataset. a Ranked dot plot indicating the risk score distribution and the ovarian cancer patients’ survival status. b Scatter dot plot indicating the risk score distribution and the ovarian cancer patients’ survival time and status. c KM analysis of the overall survival between the high and low risk groups. d Heatmap showing the expression distribution of 13 risk genes in high and low risk groups. **Figure S10.** The correlation of the CRGs and risk genes. **Figure S11.** GSEA analysis of total cohort based on risk signature. a GSEA analysis result applied the “c5.go.v7.4.symbols.gmt” as gene set. b GSEA analysis result applied the “c2.cp.kegg.v7.4.symbols.gmt” as gene set.

**Additional file 2: Table S1.** The clinicaopathological factors of the total cohort (TCGA_OV, GSE53963, GSE73614). **Table S2.** Summary of 13 recognized cuproptosis-related genes. **Table S3.** The prognostic values of 13 PRGs in patients with OV. **Table S4.** The activation states of KEGG biological pathways in different cuproptosis subtype by GSVA enrichment analysis. **Table S5.** Functional annotation of the inter-differentially expressed genes in the three cuproptosis clusters. **Table S6.** KEGG enrichment analysis of the inter-differentially expressed genes in the three cuproptosis clusters. **Table S7.** Prognostic analysis of 48 cuproptosis cluster-related genes using a univariate Cox regression model in OV patients. **Table S8.** The gene name and correlation coefficients after LASSO and multicox analysis for risk signatrure construction. **Table S9.** The estimated IC50 values for the chemotherapeutic and targeted therapeutic drugs between the two risk groups.

## Data Availability

The datasets analyzed for this study can be found in the TCGA (https://portal.gdc.cancer.gov/), GEO (https://www.ncbi.nlm.nih.gov/geo/), and TCPA (https://www.tcpaportal.org/tcpa/). Further inquiries can be directed to the corresponding author.
