# Supplementary material for: Molecular subtypes based on cuproptosis-related genes and tumor microenvironment infiltration characterization in ovarian cancer
Source: Cancer Cell Int. 2022 Oct 28;22:328. doi: 10.1186/s12935-022-02756-y (PMC9617300; doi:10.1186/s12935-022-02756-y)
Supplement: Supplementary file 1 — Additional file 1: Figure S1. The Kaplan-Meier plots of 9 prognostic CRGs in ovarian cancer. Figure S2. Unsupervised clustering of cuproptosis-related genes and Consensus matrix heatmaps for k = 2,4-9, as well as the consensus cumulative distribution function (CDF) curve for 2-9 curves. Figure S3. External validation for unsupervised clustering of cuproptosis-related genes in GSE32062. a Unsupervised clustering of cuproptosis-related genes in GSE32062, showing the consensus matrix heatmaps for k = 2-5; b Kaplan–Meier curves for overall survival of 260 OC patients (GSE32062) with three cuproptosis subtypes, the significant differences were observed among the three subtypes (log-rank test, p =0.0329). Figure S4. Forest plot of 48 prognostic genes after uniCox analysis of the DEGs (all P<0.05). Figure S5. Unsupervised clustering of 48 prognostic DEGs and Consensus matrix heatmaps for k = 3–9, as well as the consensus cumulative distribution function (CDF) curve for 2–9 curves. Figure S6. Validation of risk score in testing and total group. a Ranked dot plot indicating the risk score distribution and the ovarian cancer patients’ survival status. b Scatter dot plot indicating the risk score distribution and the ovarian cancer patients’ survival time and status. c Heatmap showing the expression distribution of 13 risk genes in high and low risk groups. d KM analysis of the overall survival between the high and low risk groups. e The unicox analysis for identifying the independent risk factors. f The multicox analysis for identifying the independent risk factors. Figure S7. Validation of risk score in GSE53963 dataset. a Ranked dot plot indicating the risk score distribution and the ovarian cancer patients’ survival status. b Scatter dot plot indicating the risk score distribution and the ovarian cancer patients’ survival time and status. c Heatmap showing the expression distribution of 13 risk genes in high and low risk groups. d KM analysis of the overall survival betwee [file 12935_2022_2756_MOESM1_ESM.docx]

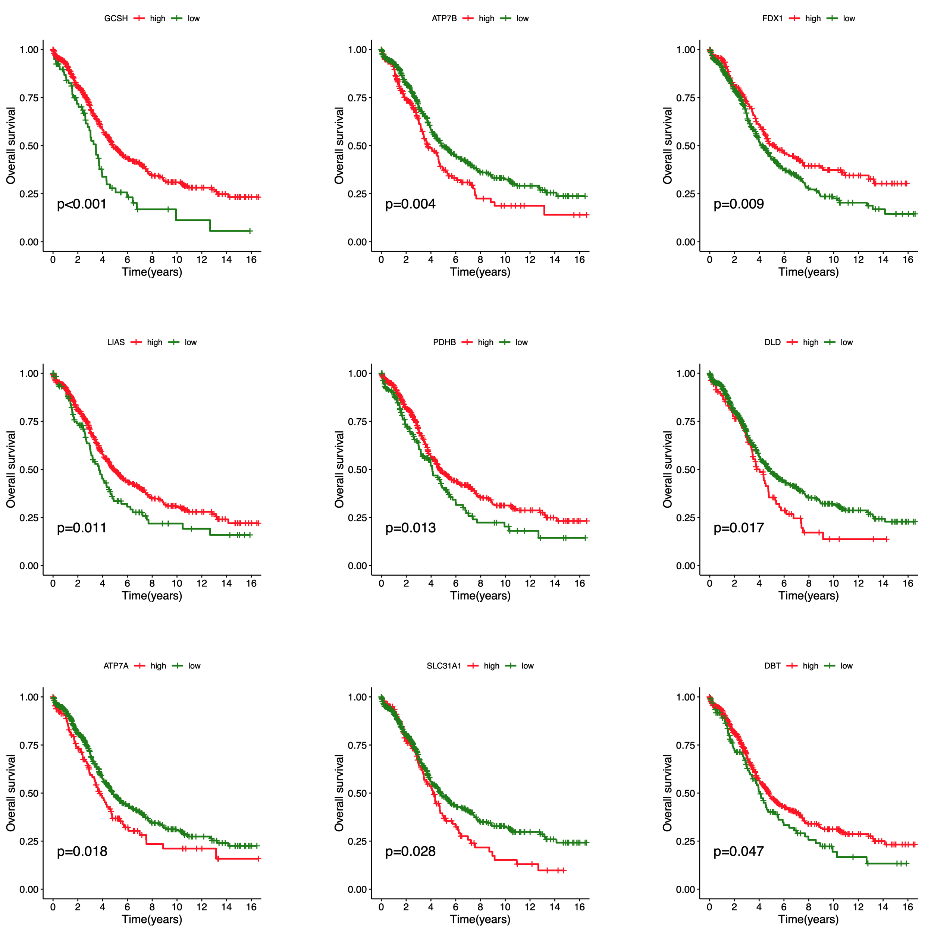


Figure S1. The Kaplan-Meier plots of 9 prognostic CRGs in ovarian cancer


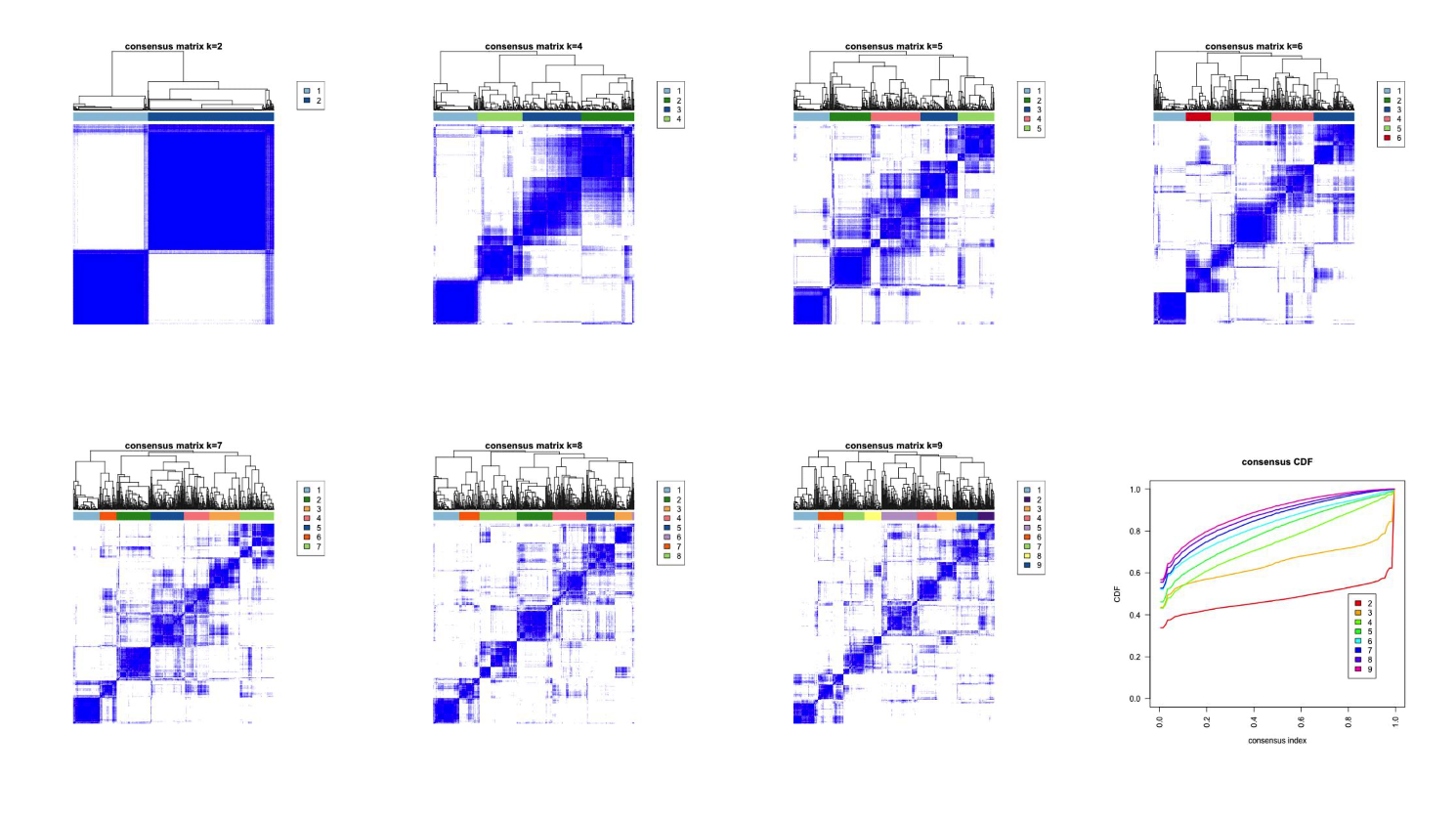


Figure S2. Unsupervised clustering of cuproptosis-related genes and Consensus matrix heatmaps for k = 2,4-9, as well as the consensus cumulative distribution function (CDF) curve for 2-9 curves.


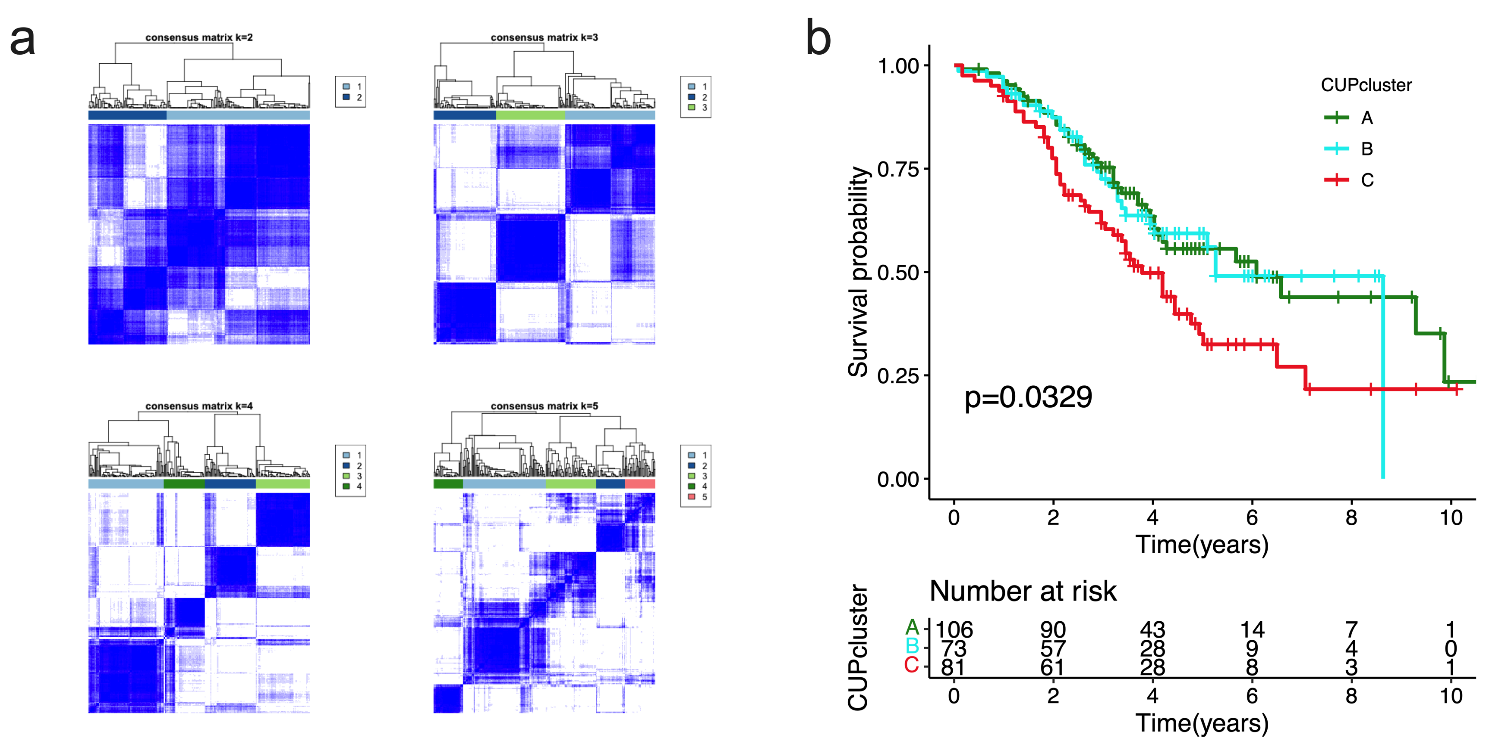


Figure S3. External validation for unsupervised clustering of cuproptosis-related genes in GSE32062.

1. Unsupervised clustering of cuproptosis-related genes in GSE32062, showing the consensus matrix heatmaps for k = 2-5;
2. Kaplan–Meier curves for overall survival of 260 OC patients (GSE32062) with three cuproptosis subtypes, the significant differences were observed among the three subtypes (log-rank test, p =0.0329).


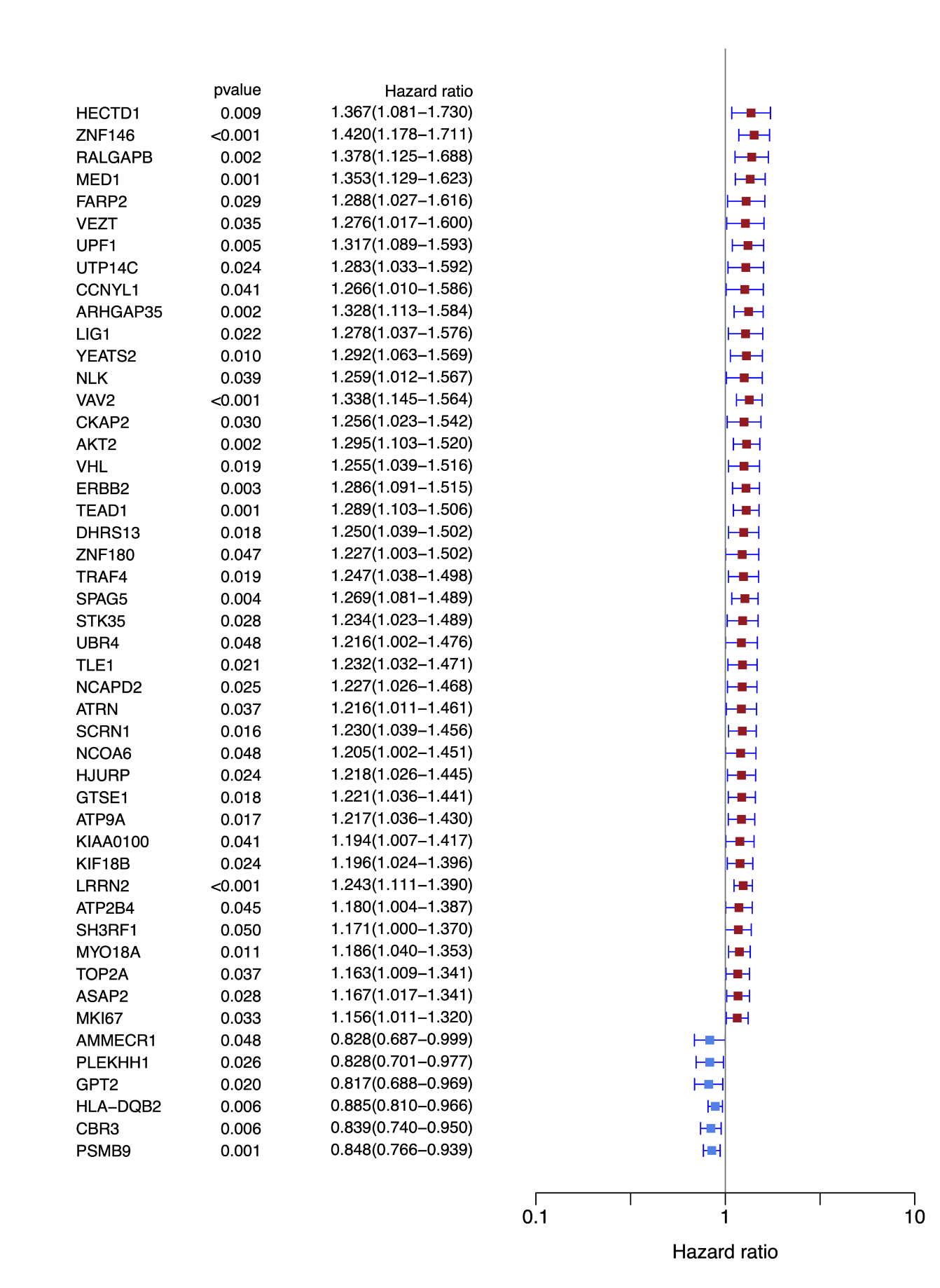


Figure S4. Forest plot of 48 prognostic genes after uniCox analysis of the DEGs (all P<0.05)


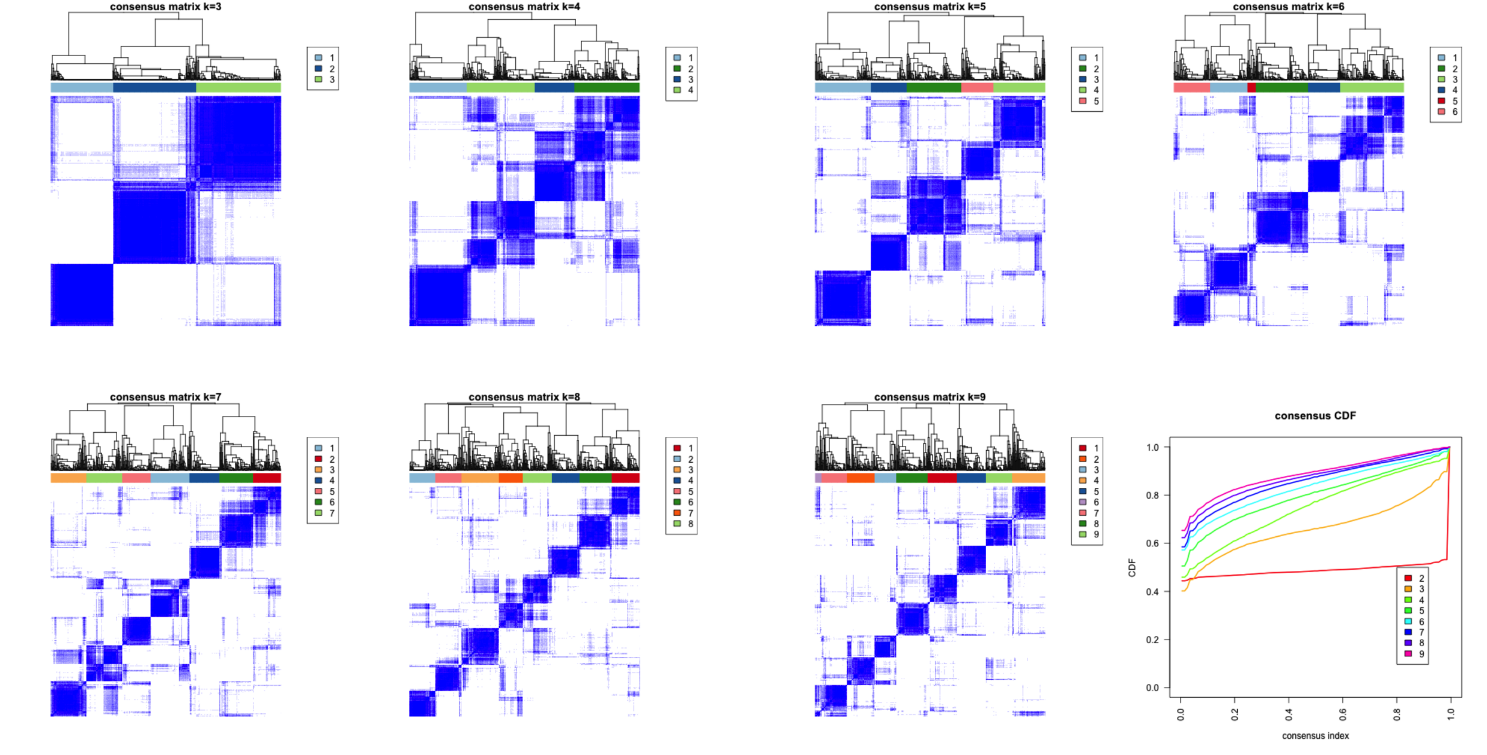


Figure S5. Unsupervised clustering of 48 prognostic DEGs and Consensus matrix heatmaps for k = 3-9, as well as the consensus cumulative distribution function (CDF) curve for 2-9 curves.


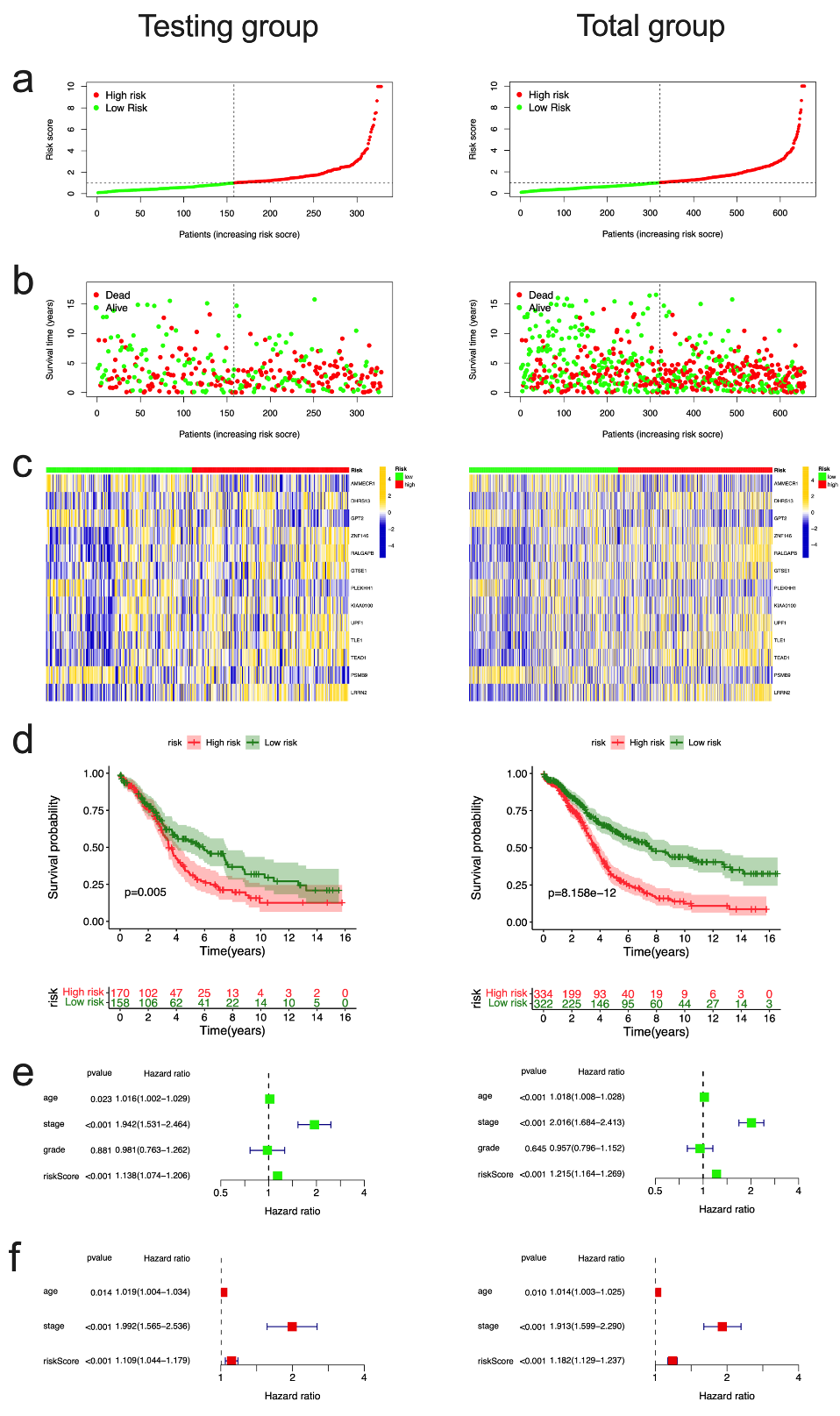


Figure S6. Validation of risk score in testing and total group.

1. Ranked dot plot indicating the risk score distribution and the ovarian cancer patients’ survival status.
2. Scatter dot plot indicating the risk score distribution and the ovarian cancer patients’ survival time and status.
3. Heatmap showing the expression distribution of 13 risk genes in high and low risk groups.
4. KM analysis of the overall survival between the high and low risk groups.
5. The unicox analysis for identifying the independent risk factors.
6. The multicox analysis for identifying the independent risk factors.


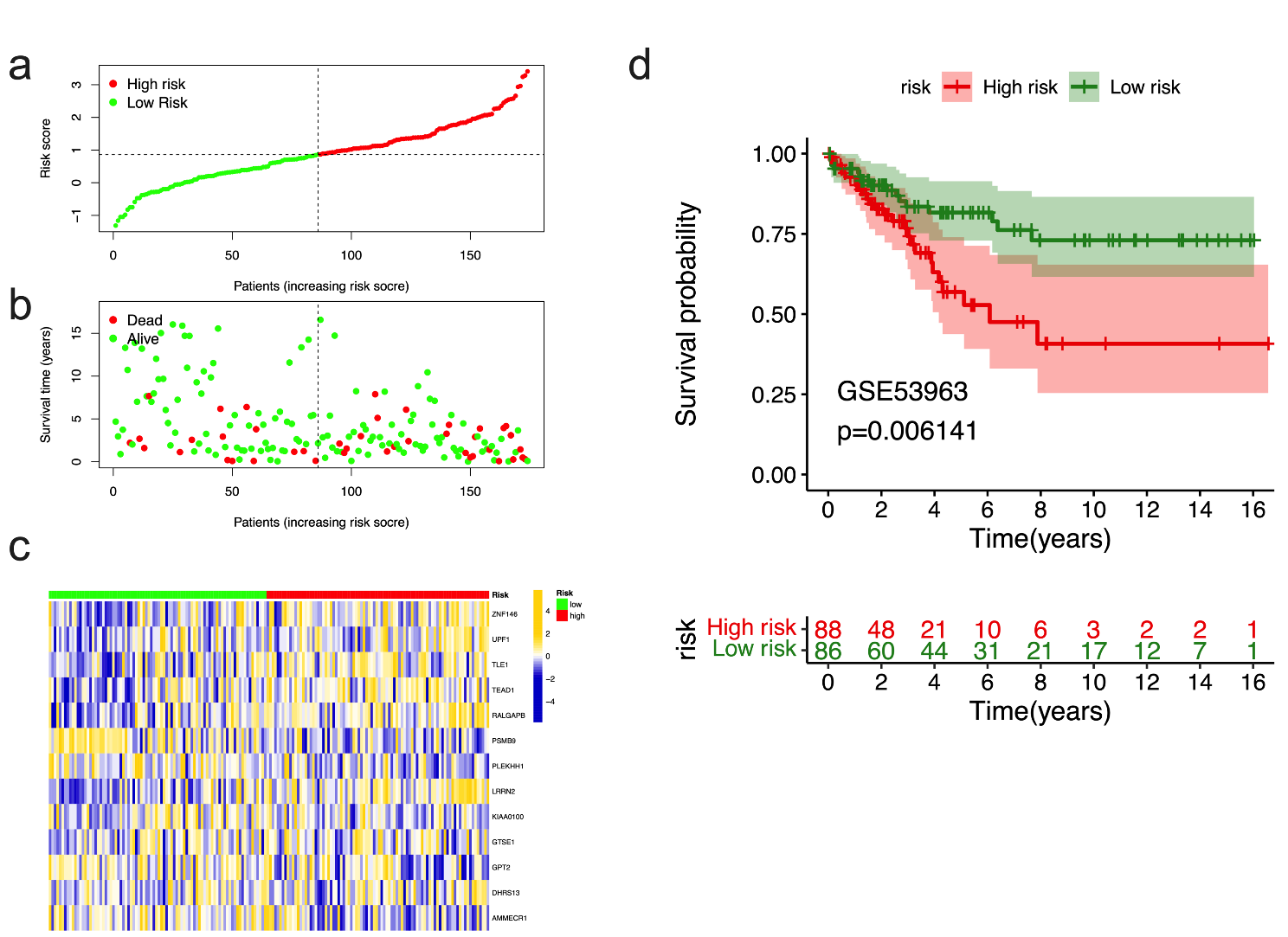


Figure S7. Validation of risk score in GSE53963 dataset.

1. Ranked dot plot indicating the risk score distribution and the ovarian cancer patients’ survival status.
2. Scatter dot plot indicating the risk score distribution and the ovarian cancer patients’ survival time and status.
3. Heatmap showing the expression distribution of 13 risk genes in high and low risk groups.
4. KM analysis of the overall survival between the high and low risk groups.


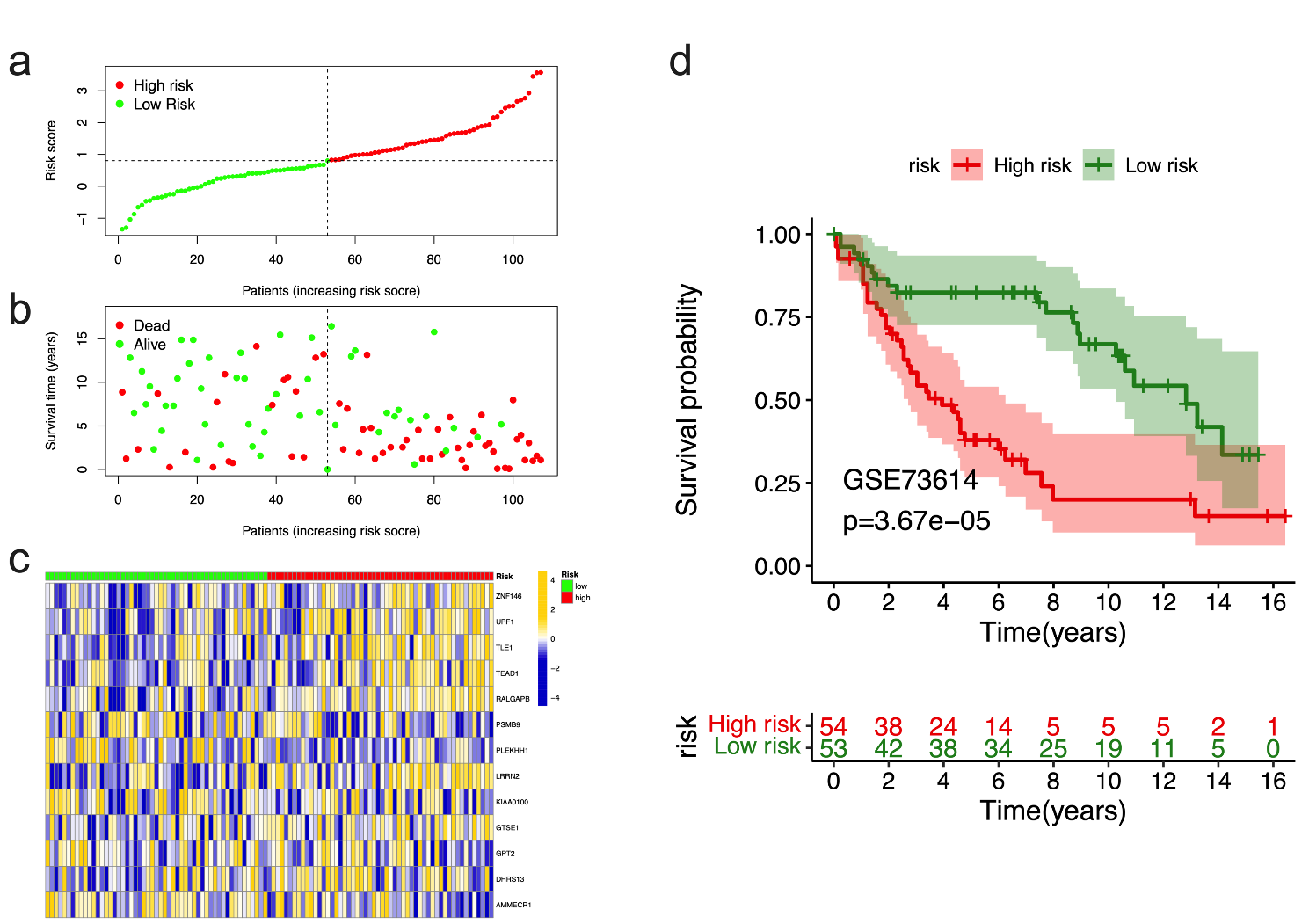


Figure S8. Validation of risk score in GSE74614 dataset.

1. Ranked dot plot indicating the risk score distribution and the ovarian cancer patients’ survival status.
2. Scatter dot plot indicating the risk score distribution and the ovarian cancer patients’ survival time and status.
3. Heatmap showing the expression distribution of 13 risk genes in high and low risk groups.
4. KM analysis of the overall survival between the high and low risk groups.


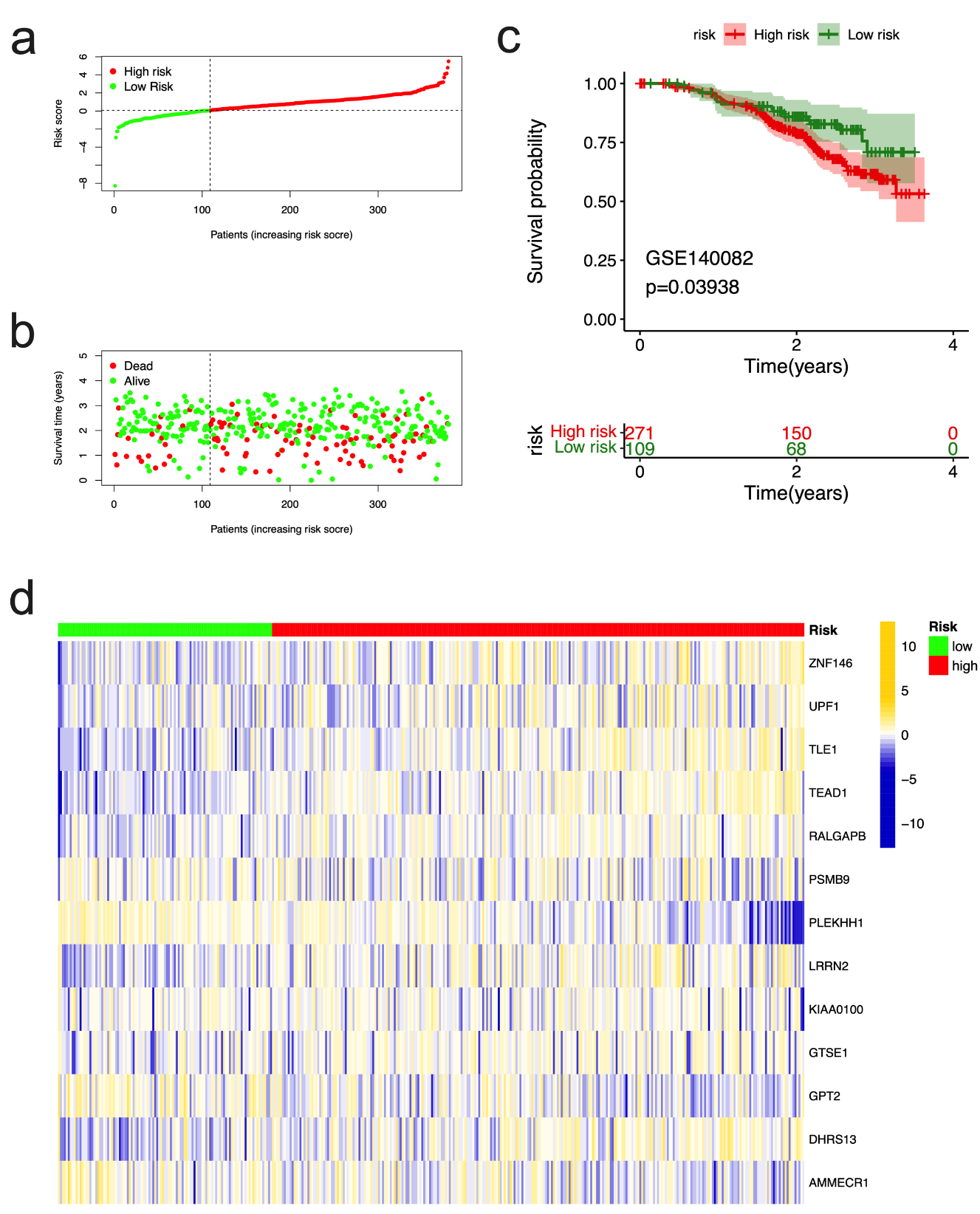


Figure S9. Validation of risk score in GSE140082 dataset.

1. Ranked dot plot indicating the risk score distribution and the ovarian cancer patients’ survival status.
2. Scatter dot plot indicating the risk score distribution and the ovarian cancer patients’ survival time and status.
3. KM analysis of the overall survival between the high and low risk groups.
4. Heatmap showing the expression distribution of 13 risk genes in high and low risk groups.


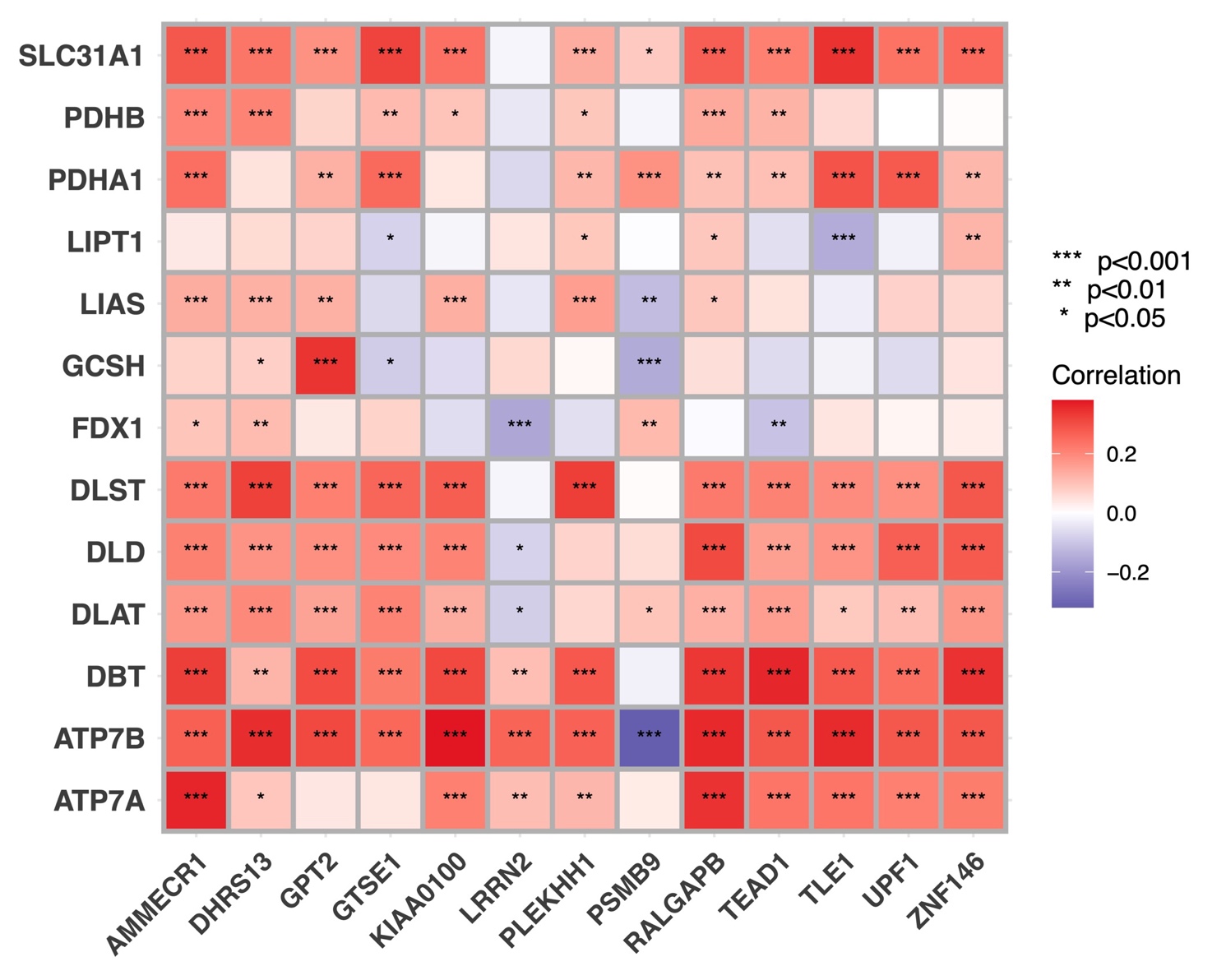


Figure S10. The correlation of the CRGs and risk genes.


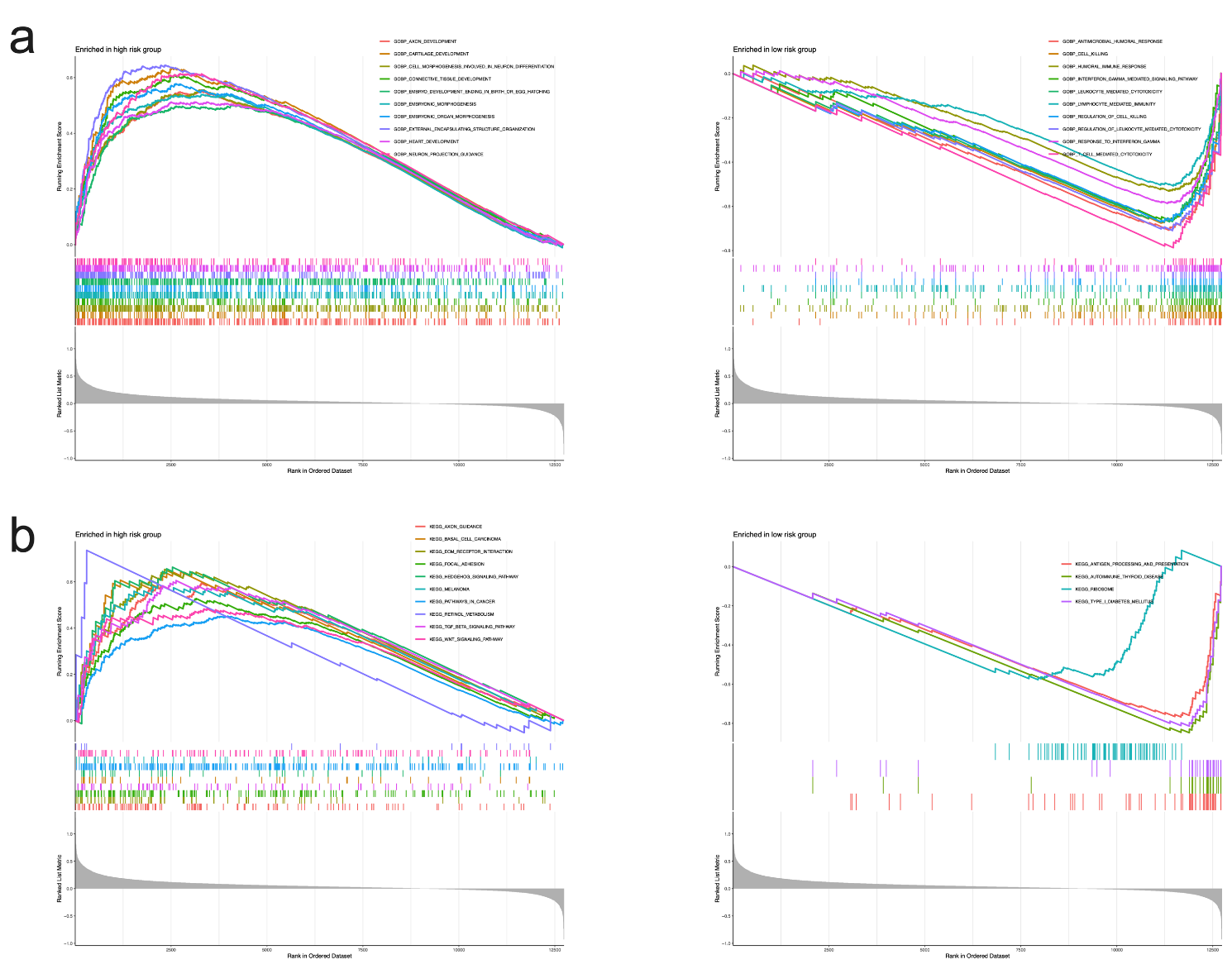


Figure S11. GSEA analysis of total cohort based on risk signature

1. GSEA analysis result applied the “c5.go.v7.4.symbols.gmt” as gene set.
2. GSEA analysis result applied the “c2.cp.kegg.v7.4.symbols.gmt” as gene set.
